# On Optimal and Quantum Code Construction from Cyclic Codes over F_q_*PQ* with Applications

**DOI:** 10.3390/e25081161

**Published:** 2023-08-02

**Authors:** Shakir Ali, Amal S. Alali, Pushpendra Sharma, Kok Bin Wong, Elif Segah Öztas, Mohammad Jeelani

**Affiliations:** 1 Department of Mathematics, Faculty of Science, Aligarh Muslim University, Aligarh 202002, India; shakir.ali.mm@amu.ac.in; 2 Department of Mathematical Sciences, College of Science, Princess Nourah bint Abdulrahman University, P.O. Box 84428, Riyadh 11671, Saudi Arabia; asalali@pnu.edu.sa; 3 Institute of Mathematical Sciences, University of Malaya, Kuala Lumpur 50603, Malaysia; kbwong@um.edu.my; 4 Department of Mathematics, Kamil Ozdag Science Faculty, Karamanoglu Mehmetbey University, Karaman 70100, Turkey; esoztas@kmu.edu.tr; 5 Department of Computer Application, Faculty of Science, Integral University, Lucknow 226026, India; jeelani.0018@gmail.com

**Keywords:** cyclic code, dual code, mixed alphabet code, QEC code, 94B05, 94B15, 94B60

## Abstract

The key objective of this paper is to study the cyclic codes over mixed alphabets on the structure of $F_{q}PQ$, where $P=\frac{F_{q}\left[v\right]}{\langle v^{3}-\alpha_{2}^{2}v\rangle }$ and $Q=\frac{F_{q}\left[u,v\right]}{\langle u^{2}-\alpha_{1}^{2},v^{3}-\alpha_{2}^{2}v\rangle }$ are nonchain finite rings and $\alpha_{i}$ is in $F_{q}/\left\{0\right\}$ for i∈{1,2}, where $q=p^{m}$ with m≥1 is a positive integer and *p* is an odd prime. Moreover, with the applications, we obtain better and new quantum error-correcting (QEC) codes. For another application over the ring *P*, we obtain several optimal codes with the help of the Gray image of cyclic codes.

## 1. Introduction

The most significant families of cyclic codes were first introduced and studied by Prange [1] and Sloane-Thompson [2]. These codes are extensively used because of their robust algebraic structure and simplicity of usage. In recent years, there has been a rapid expansion of research on cyclic codes over finite rings, following the notable work of Hammons et al. [3]. The literature extensively delves into the exploration of cyclic codes and their constructions across various finite rings, such as applications in constructing minimal codes in [4] and projective two-weight codes in [5]. Recently, Pereira and Mancini have given a general method to construct EAQEC codes from cyclic codes in [6]. A particular area of interest in recent years has been the study of codes over mixed alphabets. This research direction was initiated by Brouwer et al. [7] in 1998, where they began investigating linear codes over mixed alphabets. Specifically, the authors focused on describing $Z_{2}$-submodules over $Z_{r^{2}}Z_{s^{3}}$ for mixed alphabet codes. Borges et al. [8] made significant contributions to this field by discovering $Z_{2}Z_{4}$-additive codes and their associated $Z_{2}Z_{4}$-linear codes. Notably, extensive studies have been conducted on additive codes, with significant research contributions in [9,10,11,12]. Moreover, the additive codes, additive cyclic codes, and the additive quasi-cyclic codes over different mixed alphabets have also been intensely studied, for example, $Z_{2}Z_{2}\left[u\right]$-additive codes [13], $Z_{p}Z_{p^{k}}$-additive codes [14], $Z_{2}Z_{2}\left[u\right]$-cyclic and constacyclic [15], $Z_{2}\left(Z_{2}+uZ_{2}\right)$-additive cyclic codes [16]. Borges et al. [8] explored double cyclic codes over $Z_{2}$. Recently, Gao et al. have studied hulls of double cyclic codes over $Z_{2}$, and obtained some good quantumcodes from hulls in [17]. Gao et al. [18] generalized double cyclic codes over $Z_{4}$. The triple cyclic codes over $Z_{2}$ were introduced by Mostafanasab [19] and extended this double cyclic code structure. Recently, $Z_{2}Z_{2}Z_{4}$-additive cyclic codes and $Z_{2}Z_{4}Z_{8}$-cyclic codes were separately explored by Wu et al. [20], and Aydogdu-Gursoy [21], respectively. Moreover, the *P* and *Q* used in this paper are finite nonchain rings actually. As we know, there are many papers on quantum codes over finite nonchain rings [22,23,24]. Researchers primarily concentrated on investigating the structural properties of mixed alphabet codes in all of the works, including generator matrices, parity check matrices, generator polynomials, minimal generating sets, generator polynomials for dual codes, etc. In 2020, Dinh et al. [25] delivered quantum and LCD code construction over mixed alphabets. In 2022, Ashraf et al. [26] obtained quantum codes over mixed alphabets.

Motivated by the above study, in this paper, we describe cyclic codes, new quantum error-correcting (QEC) codes and several optimal codes over mixed alphabets. Firstly, we provide linear and cyclic codes over $F_{q}PQ$, where $F_{q}$ are the finite fields with *q* elements, $P=F_{q}\left[v\right]/\langle v^{3}-\alpha_{2}^{2}v\rangle$ and $Q=F_{q}\left[u,v\right]/\langle u^{2}-\alpha_{1}^{2},v^{3}-\alpha_{2}^{2}v\rangle$, where $\alpha_{1}$ and $\alpha_{2}$ are the nonzero elements of $F_{q}$. Section 2 presents some basic definitions, the construction of cyclic codes over $F_{q}PQ$, and some important structural properties over $F_{q}PQ$. Section 3 describes Gray images and linear codes over *P* and *Q*. Further, we define a Gray map with the help of a matrix. In Section 4, like Section 3, we define the Gray map and linear codes over $F_{q}PQ$. Section 5 discusses the structural properties of cyclic codes over *P*, *Q*, $F_{q}PQ$ and describes quantum error-correcting (QEC) codes and their construction over $F_{q}PQ$. Finally, in Section 6, we discuss some applications of cyclic codes over mixed alphabets and provide the conclusion of our results.

## 2. Preliminaries

Let *m* be a positive integer, *p* be an odd prime, and *q* be an odd prime power such that $q=p^{m}$. Next, let $F_{q}$ be a finite field with *q* elements having characteristic *p*. Our construction depends on $F_{q}PQ$, where *P* and *Q* are the commutative, nonchain, semi-local ring. We begin with some key remarks and basic definitions as follows:

**Remark 1.**  *Let R be a local ring. Then, the following conditions are equivalent:*

*(i).*  *R has a unique maximal left ideal.*
*(ii).*  *R has a unique maximal right ideal.*
*(iii).*  *The sum of any two nonunit elements of R is also a nonunit as well as 0≠1.*
*(iv).*  *If x is an arbitrary element of R, then x or 1−x is unit as well as 0≠1.*

**Remark 2.**  *In the case of a commutative ring, R contains a unique maximal ideal if and only if it is local.*

**Definition 1.**  *Let $x,y\in F_{q}^{n}$, the Hamming distance between two vectors $x=x_{1}\ldots x_{n}$ and $y=y_{1}\ldots y_{n}$ be defined to be the number of places at which they differ and be denoted by d(x,y).*

**Definition 2.**  *The Hamming weight of a vector $x=x_{1}x_{2}\ldots x_{n}$ is defined to be the number of nonzero coordinates $x_{i}$ in x and is denoted by $w_{H}\left(x\right)$.*

**Definition 3.**  *Each element of code E is referred to as a codeword, and a code of length n over R is said to be linear if it is an R-submodule of $R^{n}.$*

**Definition 4.**  *A code E is said to be self-orthogonal if $E\subseteq E^{⊥}$, self-dual if $E=E^{⊥}$, and dual containing if $E^{⊥}\subseteq E$.*

**Definition 5**  ([27])**.** *A code E is a cyclic code of length n over R if it is linear and every cyclic shift of each codeword is also in E, i.e., $\sigma \left(c\right)=\left(E_{n-1},\;E_{0},\;E_{1},\;\ldots ,\;E_{n-2}\right)\in E$, whenever $c=(E_{n-1},\;E_{0},\;\ldots ,\;E_{n-2})\in E$. The operator σ is known as cyclic shift.*

By using the properties of cyclic code over the finite commutative nonchain ring, we can define cyclic code over $F_{q}PQ$. Clearly, the ring *P* can be expressed as $P=F_{q}+vF_{q}+v^{2}F_{q}$, where $v^{3}=\alpha_{2}^{2}v$, the set $\left\{1,v,v^{2}\right\}$ is a set of basis elements of *P*, and we denote the basis elements of *P* as follows: $\Delta_{1}=1,\Delta_{2}=v,\Delta_{3}=v^{2},$ and every element of ring *P* is of the form $p_{r}=a_{1}+va_{2}+v^{2}a_{3}$, for $a_{1},a_{2},a_{3}\in F_{q}$. Orthogonal idempotents of this ring is given as follows:

$$E_{1}=\left(\alpha_{2}^{2}-v^{2}\right),$$

$$E_{2}=\frac{\left(\alpha_{2}v+\alpha_{2}^{2}v^{2}\right)}{2},$$

and

$$E_{3}=\frac{\left(-\alpha_{2}v+\alpha_{2}^{2}v^{2}\right)}{2}.$$

It is straightforward to see that $E_{i}^{2}=E_{i},\;E_{i}E_{j}=0$ for i≠j, and $E_{1}+E_{2}+E_{3}=1$ where i,j=1,2,3. By using orthogonal idempotents $E_{1},E_{2}$ and $E_{3}$, we can write the arbitrary element $p_{r}$ of the ring *P* as $p_{r}=E_{1}p_{r_{1}}+E_{2}p_{r_{2}}+E_{3}p_{r_{3}}$, where $p_{r_{1}},p_{r_{2}},p_{r_{3}}\in F_{q}$. Similarly, the ring *Q* can be expressed as $Q=F_{q}+uF_{q}+vF_{q}+uvF_{q}+v^{2}F_{q}+uv^{2}F_{q}$, where $u^{2}=\alpha_{1}^{2}$, $v^{3}=\alpha_{2}^{2}v$, the set $\left\{1,u,v,uv,v^{2},uv^{2}\right\}$ is a set of basis elements of *Q* and we denote the basis elements of *Q* as follows $\delta_{1}=1,\delta_{2}=u$ and $\delta_{3}=v,\delta_{4}=uv,\delta_{5}=v^{2},\delta_{6}=uv^{2}$ and every element of ring *Q* is of the form $q_{r}=b_{1}+ub_{2}+vb_{3}+uvb_{4}+v^{2}b_{5}+uv^{2}b_{6}$, for $b_{1},b_{2},b_{3},b_{4},b_{5},b_{6}\in F_{q}$. Orthogonal idempotents of the ring *Q* are given as follows:

$$\zeta_{1}=\frac{\left(\alpha_{1}+u\right)\left(\alpha_{2}^{2}-v^{2}\right)}{2\alpha_{1}\alpha_{2}^{2}},$$

$$\zeta_{2}=\frac{\left(\alpha_{1}-u\right)\left(\alpha_{2}^{2}-v^{2}\right)}{2\alpha_{1}\alpha_{2}^{2}},$$

$$\zeta_{3}=\frac{\left(\alpha_{1}+u\right)\left(\alpha_{2}v+v^{2}\right)}{4\alpha_{1}\alpha_{2}^{2}},$$

$$\zeta_{4}=\frac{\left(\alpha_{1}-u\right)\left(\alpha_{2}v+v^{2}\right)}{4\alpha_{1}\alpha_{2}^{2}},$$

$$\zeta_{5}=\frac{\left(\alpha_{1}+u\right)\left(-\alpha_{2}v+v^{2}\right)}{4\alpha_{1}\alpha_{2}^{2}}$$

and

$$\zeta_{6}=\frac{\left(\alpha_{1}-u\right)\left(-\alpha_{2}v+v^{2}\right)}{4\alpha_{1}\alpha_{2}^{2}}.$$

It is easy to see that $\zeta_{i}^{2}=\zeta_{i},\;\zeta_{i}\zeta_{j}=0$ and $\zeta_{1}+\zeta_{2}+\zeta_{3}+\zeta_{4}+\zeta_{5}+\zeta_{6}=1$ where i,j=1,2,3,4,5,6 and i≠j. By using orthogonal idempotents $\zeta_{1},\zeta_{2},\zeta_{3},\zeta_{4},\zeta_{5}$ and $\zeta_{6}$, we can write the arbitrary element $q_{r}$ of the ring *Q* as $q_{r}=\zeta_{1}q_{r_{1}}+\zeta_{2}q_{r_{2}}+\zeta_{3}q_{r_{3}}+\zeta_{4}q_{r_{4}}+\zeta_{5}q_{r_{5}}+\zeta_{6}q_{r_{6}}$, where $q_{r_{1}},q_{r_{2}},q_{r_{3}},q_{r_{4}},q_{r_{5}},q_{r_{6}}\in F_{q}$. Now, we define two ring homomorphisms η and ζ as

$$\eta :Q⟶F_{q}$$

such that $\eta \left(q_{r}\right)=\eta \left(\zeta_{1}q_{r_{1}}+\zeta_{2}q_{r_{2}}+\zeta_{3}q_{r_{3}}+\zeta_{4}q_{r_{4}}+\zeta_{5}q_{r_{5}}+\zeta_{6}q_{r_{6}}\right)=q_{r_{1}}$ and

Γ:Q⟶P

such that $\Gamma \left(\zeta_{1}q_{r_{1}}+\zeta_{2}q_{r_{2}}+\zeta_{3}q_{r_{3}}+\zeta_{4}q_{r_{4}}+\zeta_{5}q_{r_{5}}+\zeta_{6}q_{r_{6}}\right)=E_{1}q_{r_{1}}+E_{2}q_{r_{2}}+E_{3}q_{r_{3}}$. For arbitrary $q_{r}\in Q$ and $\left(x,y,z\right)\in F_{q}PQ$, we define *Q*-scalar multiplication on $F_{q}PQ$ by:

$$⧫:Q\times F_{q}PQ⟶F_{q}PQ$$

such that

$$q_{r}⧫\left(x,y,z\right)=\left(\eta \left(q_{r}\right)\left(x\right),\;\Gamma \left(q_{r}\right)\left(y\right),\;q_{r}z\right).$$

This multiplication is well defined and we can extend this multiplication over $F_{q}^{\alpha }\times P^{\beta }\times Q^{\gamma }$ as

$$⧫:Q\times \left(F_{q}^{\alpha }\times P^{\beta }\times Q^{\gamma }\right)⟶F_{q}^{\alpha }\times P^{\beta }\times Q^{\gamma }$$

such that $q_{r}⧫l=(\eta \left(q_{r}\right)x_{0},\eta \left(q_{r}\right)x_{1},\ldots ,\eta \left(q_{r}\right)x_{\alpha -1},\Gamma \left(q_{r}\right)y_{0},\Gamma \left(q_{r}\right)y_{1},\ldots ,\Gamma \left(q_{r}\right)y_{\beta -1},q_{r}z_{0},q_{r}z_{1},$$\ldots ,q_{r}z_{\gamma -1})$, where $q_{r}\in Q$ and $l=\left(x_{0},x_{1},\ldots ,x_{\alpha -1},y_{0},\ldots ,y_{\beta -1},z_{0},z_{1},\ldots ,z_{\gamma -1}\right)\in F_{q}^{\alpha }\times P^{\beta }\times Q^{\gamma }.$

In view of this scalar multiplication, $F_{q}^{\alpha }\times P^{\beta }\times Q^{\gamma }$ forms a Q-module.

**Definition 6.**  *A nonempty subset E of $F_{q}^{\alpha }\times P^{\beta }\times Q^{\gamma }$ is a $F_{q}PQ$-linear code of length (α+β+γ) if E is a Q-submodule of $F_{q}^{\alpha }\times P^{\beta }\times Q^{\gamma }$.*

Let $l=(x_{0},x_{1},\ldots ,x_{\alpha -1},y_{0},\ldots ,y_{\beta -1},z_{0},z_{1},\ldots ,z_{\gamma -1})$ and $l^{\prime }=(x_{0}^{\prime },x_{1}^{\prime },\ldots ,x_{\alpha -1}^{\prime },y_{0}^{\prime },\ldots ,$$y_{\beta -1}^{\prime },z_{0}^{\prime },z_{1}^{\prime },\ldots ,z_{\gamma -1}^{\prime })$, where $l,\;l^{\prime }\in F_{q}^{\alpha }\times P^{\beta }\times Q^{\gamma }$. After this, we also define inner product of *l* and $l^{\prime }$ as

$$l\cdot l^{\prime }=\sum_{i=0}^{\alpha -1}x_{i}x_{i}^{\prime }+\sum_{j=0}^{\beta -1}y_{j}y_{j}^{\prime }+\sum_{k=0}^{\gamma -1}z_{k}z_{k}^{\prime }\in Q.$$

Here, the dual of E, i.e., $E^{⊥}=\left\{l^{\prime }\in F_{q}^{\alpha }\times P^{\beta }\times Q^{\gamma }\;|\;l\cdot l^{\prime }=0,\;\forall \;l\in E\right\}$.

**Definition 7.**  *A linear code E is a $F_{q}PQ$-cyclic code of length (α+β+γ) if every cyclic shift of E is also in E, i.e., $\sigma \left(c\right)=\left(x_{\alpha -1},x_{0},x_{1},\ldots ,x_{\alpha -2},p_{\beta -1},p_{0},p_{1},\ldots ,p_{\beta -2},q_{\gamma -1},q_{0},q_{1},\ldots ,q_{\gamma -2}\right)\in E,\;\forall \;c\in E$, where $c=(x_{0},x_{1},\ldots ,x_{\alpha -1},p_{0},\ldots ,p_{\beta -1},q_{0},q_{1},\ldots ,q_{\gamma -1})$ and σ(c) is a cyclic shift of E.*

**Proposition 1.**  *Suppose E is a $F_{q}PQ$-cyclic code of length (α+β+γ). Then, the dual of E is a also $F_{q}PQ$-cyclic code of length (α+β+γ).*

**Proof **  Suppose E is a $F_{q}PQ$-cyclic code of length (α+β+γ), and next, let us consider that $l^{\prime }\in E^{⊥}$ such that $l^{\prime }=\left(x_{0}^{\prime },x_{1}^{\prime },\ldots ,x_{\alpha -1}^{\prime },p_{0}^{\prime },p_{1}^{\prime },\ldots ,p_{\beta -1}^{\prime },q_{0}^{\prime },q_{1}^{\prime },\ldots ,q_{\gamma -1}^{\prime }\right)$, and also, we take lcm(α,β,γ)=t and $l=(x_{0},x_{1},\ldots ,x_{\alpha -1},p_{0},p_{1},\ldots ,p_{\beta -1},q_{0},q_{1},\ldots ,q_{\gamma -1})\in E$. Then, we will show that $\sigma \left(l^{\prime }\right)=\left(x_{\alpha -1}^{\prime },x_{0}^{\prime },x_{1}^{\prime },\ldots ,x_{\alpha -2}^{\prime },p_{\beta -1}^{\prime },p_{0}^{\prime },p_{1}^{\prime },\ldots ,p_{\beta -2}^{\prime },q_{\gamma -1}^{\prime },q_{0}^{\prime },q_{1}^{\prime },\ldots ,q_{\gamma -2}^{\prime }\right)\in E^{⊥}.$ From above described inner product, we have

$$\begin{array}{ccc} l.\sigma (l^{\prime }) & = & \left(x_{0}x_{\alpha -1}^{\prime }+x_{1}x_{0}^{\prime }+\ldots +x_{\alpha -1}x_{\alpha -2}^{\prime }\right)+\left(p_{0}p_{\beta -1}^{\prime }+p_{1}p_{0}^{\prime }+\ldots +p_{\beta -1}p_{\beta -2}^{\prime }\right)+\\ & \; & (q_{0}q_{\gamma -1}^{\prime }+q_{1}q_{0}^{\prime }+\ldots +q_{\gamma -1}q_{\gamma -2}^{\prime }). \end{array}$$

Since E is a $F_{q}PQ$-cyclic code and lcm(α,β,γ)=t.

$$\sigma^{t-1}\left(l\right)=\left(x_{1},\ldots ,x_{\alpha -1},x_{0},p_{1},\ldots ,p_{\beta -1},p_{0},q_{1},\ldots ,q_{\gamma -1},q_{0}\right).$$

Now, we take the inner product of $\sigma^{t-1}\left(l\right)$ and $l^{\prime }$, we have

$$\sigma^{t-1}\left(l\right)\cdot l^{\prime }=0,$$

where

$$\begin{array}{ccc} \sigma^{t-1}\left(l\right).l^{\prime } & = & \left(x_{1}x_{0}^{\prime }+x_{2}x_{1}^{\prime }+\ldots +x_{0}x_{\alpha -1}^{\prime }\right)+\left(p_{1}p_{0}^{\prime }+p_{2}p_{1}^{\prime }+\ldots +p_{0}p_{\beta -1}^{\prime }\right)+\\ & \; & \left(q_{1}q_{0}^{\prime }+q_{2}q_{1}^{\prime }+\ldots +q_{0}q_{\gamma -1}^{\prime }\right) \end{array}$$

On comparing the coefficients of both sides, we have

$$\begin{array}{ccc} x_{1}x_{0}^{\prime }+x_{2}x_{1}^{\prime }+\ldots +x_{0}x_{\alpha -1}^{\prime } & = & 0\\ p_{1}p_{0}^{\prime }+p_{2}p_{1}^{\prime }+\ldots +p_{0}p_{\beta -1}^{\prime } & = & 0\\ q_{1}q_{0}^{\prime }+q_{2}q_{1}^{\prime }+\ldots +q_{0}q_{\gamma -1}^{\prime } & = & 0 \end{array}$$

we obtain

$$l\cdot \sigma (l^{\prime })=0.$$

Thus, $\sigma \left(l^{\prime }\right)\in E^{⊥}.$ This shows that $E^{⊥}$ is a $F_{q}PQ$-cyclic code of length (α+β+γ). □

Let

$$Q_{\alpha +\beta +\gamma }=\frac{F_{q}\left[x\right]}{\langle x^{\alpha }-1\rangle }\times \frac{P[x]}{\langle x^{\beta }-1\rangle }\times \frac{Q[x]}{\langle x^{\gamma }-1\rangle }$$

and $f=\left(a_{0},a_{1},\ldots ,a_{\alpha -1},b_{0},b_{1},\ldots ,b_{\beta -1},c_{0},c_{1},\ldots ,c_{\gamma -1}\right)\in F_{q}^{\alpha }\times P^{\beta }\times Q^{\gamma }$. Let *f* be an arbitrary element of $Q_{\alpha +\beta +\gamma }$, and then *f* can be identified as

$$\begin{array}{ccc} f(x) & = & (a_{0}+a_{1}x+a_{2}x^{2}+\ldots +a_{\alpha -1}x^{\alpha -1},b_{0}+b_{1}x+b_{2}x^{2}+\ldots +b_{\beta -1}x^{\beta -1},c_{0}+c_{1}x+c_{2}x^{2}\\ & \; & +\ldots +c_{\gamma -1}x^{\gamma -1})\\ & = & (a(x),b(x),c(x)). \end{array}$$

This gives bijective mapping between $F_{q}^{\alpha }\times P^{\beta }\times Q^{\gamma }$ and $Q_{\alpha +\beta +\gamma }$. Next, let us consider that $g\left(x\right)=q_{0}+q_{1}x+q_{2}x^{2}+\ldots +q_{n}x^{n}\in Q\left[x\right]$ and $\left(a\left(x\right),b\left(x\right),c\left(x\right)\right)\in Q_{\alpha +\beta +\gamma }$. With the help of previously defined Q-scalar multiplication, we induce the multiplication ⨀ in $Q_{\alpha +\beta +\gamma }$ as g(x)⨀(a(x),b(x),c(x))=(η(g(x))a(x),Γ(g(x))b(x),g(x)c(x), where $\eta \left(g\left(x\right)\right)=\eta \left(q_{0}\right)+\eta \left(q_{1}\right)x+\ldots +\eta \left(q_{n}\right)x^{n}$ and $\Gamma \left(g\left(x\right)\right)=\Gamma \left(q_{0}\right)+\Gamma \left(q_{1}\right)x+\ldots +\Gamma \left(q_{n}\right)x^{n}$. It is simple to demonstrate that $Q_{\alpha +\beta +\gamma }$ makes an Q[x]-submodule with respect to multiplication ⨀.

**Proposition 2**  ([25])**.** *A code E is a $F_{q}PQ$-cyclic code of length (α+β+γ) if and only if E is a Q[x]-submodule of $Q_{\alpha +\beta +\gamma }$.*

## 3. Linear Codes and Gray Images over *P* and *Q*

In this part, we study the linear codes over *P* and *Q* as well as Gray maps. We construct Gray maps with the help of matrices. Gray maps are more intuitive and give better results. We see that *P* is a semi-local, commutative, and nonchain ring. An element $p_{r}$ of *P* is of the form $p_{r}=a_{1}+va_{2}+v^{2}a_{3}$ such that $v^{3}=\alpha_{2}^{2}v$, where $a_{1},a_{2},a_{3}\in F_{q}$.

In view of Chinese Remainder Theorem, it is clear to observe that $P=E_{1}F_{q}⊕E_{2}F_{q}⊕E_{3}F_{q}$, *P* is a semi-local, commutative, and nonchain ring, and each $p_{r}$ has representation $p_{r}=\sum_{i=1}^{3}\Delta_{i}a_{i}=\sum_{i=1}^{3}E_{i}p_{r_{i}}$, where $a_{i}$, $p_{r_{i}}$$\in F_{q}$, for i=1,2,3. We define the Gray map

(1) $$\varphi_{P}:P⟶F_{q}^{3}$$

by $\varphi_{P}\left(p_{r}\right)=\varphi_{P}\left(E_{1}p_{r_{1}}+E_{2}p_{r_{2}}+E_{3}p_{r_{3}}\right)=\left(p_{r_{1}},p_{r_{2}},p_{r_{3}}\right)A_{1}=eA_{1},$ where $A_{1}\in GL_{3}\left(F_{q}\right)$ is a fixed matrix and $GL_{3}\left(F_{q}\right)$ is the linear group of all 3×3 invertible matrices over the field $F_{q}$ such that $A_{1}A_{1}^{T}\;=\;\epsilon I_{3\times 3},$ where $A_{1}^{T}$ is the transpose of $A_{1}$ and $\epsilon \in F_{q}\setminus \left\{0\right\}$. Here, we use e for the vector $\left(p_{r_{1}},p_{r_{2}},p_{r_{3}}\right)$. With the orthogonal idempotent, we have $P=E_{1}F_{q}⊕E_{2}F_{q}⊕E_{3}F_{q}$. Every element $p_{r}\in P$ can be uniquely expressed as $p_{r}=E_{1}p_{r_{1}}+E_{2}p_{r_{2}}+E_{3}p_{r_{3}}$, where $p_{r_{i}}\in F_{q}$ and 1≤i≤3.

The above-described map (1) can be extended as

$$\varphi_{P}:P^{\beta }⟶F_{q}^{3\beta }$$

component-wise as $\left(p_{0},p_{1},\ldots ,p_{\beta -1}\right)⟶(\left(p_{r_{0,1}},p_{r_{0,2}},p_{r_{0,3}}\right)A_{1},\left(p_{r_{1,1}},p_{r_{1,2}},p_{r_{1,3}}\right)A_{1},\ldots ,(p_{r_{\beta -1,1}},$$p_{r_{\beta -1,2}},p_{r_{\beta -1,3}})A_{1})=e_{0}A_{1},e_{1}A_{1},e_{2}A_{1},\ldots ,e_{\beta -1}A_{1}$, here we take $p_{r}=\left(p_{0},p_{1},\ldots ,p_{\beta -1}\right)$ and $p_{i}=E_{1}p_{r_{i,1}}+E_{2}p_{r_{i,2}}+E_{3}p_{r_{i,3}}\in P$, where i=0,1,2,…,β−1.We define $w_{L}\left(p_{i}\right)=w_{H}\left(\varphi_{P}\left(p_{i}\right)\right)$, where $w_{L}\left(p_{i}\right)$ denotes the Lee weight of $p_{i}$ and $w_{H}$ stands for the Hamming weight over $F_{q}$. Let $B_{\beta }$ be linear code of length β over *P*; we define

$$\begin{array}{ccc} B_{\beta_{1}} & = & \{p_{r_{1}}\in F_{q}^{\beta }\;|\;\sum_{i=1}^{3}E_{i}p_{r_{i}}\in B_{\beta };\;p_{r_{2}},p_{r_{3}}\in F_{q}^{\beta }\;for\;1\leq i\leq 3\},\\ B_{\beta_{2}} & = & \{p_{r_{2}}\in F_{q}^{\beta }\;|\;\sum_{i=1}^{3}E_{i}p_{r_{i}}\in B_{\beta };\;p_{r_{1}},p_{r_{3}}\in F_{q}^{\beta }\;for\;1\leq i\leq 3\},\\ B_{\beta_{3}} & = & \{p_{r_{3}}\in F_{q}^{\beta }\;|\;\sum_{i=1}^{3}E_{i}p_{r_{i}}\in B_{\beta };\;p_{r_{1}},p_{r_{2}}\in F_{q}^{\beta }\;for\;1\leq i\leq 3\}. \end{array}$$

Then, $B_{\beta_{i}}$ is a linear code of length β over $F_{q}$, for i=1,2,3.

**Proposition 3.**  *The Gray map $\varphi_{P}$ is a linear, bijective and distance preserving map from $\left(P^{\beta },\;d_{L}\right)$ to $\left(F_{q}^{3\beta },\;d_{H}\right)$, where $d_{L}=d_{H}$.*

**Proof.**  Suppose $p_{r},\;p_{r}^{\prime }\in P^{\beta }$. Then, we have

$$\begin{array}{ccc} p_{r}\; & = & \;E_{1}p_{r_{1}}+E_{2}p_{r_{2}}+E_{3}p_{r_{3}}\\ p_{r}^{\prime }\; & = & \;E_{1}p_{r_{1}}^{\prime }+E_{2}p_{r_{2}}^{\prime }+E_{3}p_{r_{3}}^{\prime } \end{array}$$

$$\begin{array}{ccc} p_{r}+p_{r}^{\prime }\; & = & \;E_{1}p_{r_{1}}+E_{2}p_{r_{2}}+E_{3}p_{r_{3}}+E_{1}p_{r_{1}}^{\prime }+E_{2}p_{r_{2}}^{\prime }+E_{3}p_{r_{3}}^{\prime }\\ \varphi_{P}\left(p_{r}+p_{r}^{\prime }\right)\; & = & \;\left(p_{r_{1}}+p_{r_{1}}^{\prime },p_{r_{2}}+p_{r_{2}}^{\prime },p_{r_{3}}+p_{r_{3}}^{\prime }\right)A_{1}\\ & = & \left(p_{r_{1}},\;p_{r_{2}},\;p_{r_{3}}\right)A_{1}+\left(p_{r_{1}}^{\prime },\;p_{r_{2}}^{\prime },\;p_{r_{3}}^{\prime }\right)A_{1}\\ & = & \varphi_{P}\left(p_{r}\right)+\varphi_{P}\left(p_{r}^{\prime }\right) \end{array}$$

and we take $\mu \;\in \;F_{q}$

$$\begin{array}{ccc} \varphi_{P}\left(\mu p_{r}\right)\; & = & \;\varphi_{P}\left(E_{1}p_{r_{1}}+E_{2}p_{r_{2}}+E_{3}p_{r_{3}}\right)\\ & = & \left(\mu p_{r_{1}},\;\mu p_{r_{2}},\;\mu p_{r_{3}}\right)A_{1}\\ & = & \mu \left(p_{r_{1}},\;p_{r_{2}},\;p_{r_{3}}\right)A_{1}\\ & = & \mu \varphi_{P}\left(p_{r}\right). \end{array}$$

So, $\varphi_{P}$ is an $F_{q}$-linear map. Now, we will prove that $\varphi_{P}$ is a bijection.

Then, we have

$$\begin{array}{ccc} \varphi_{P}\left(p_{r}\right) & = & \varphi_{P}\left(p_{r}^{\prime }\right)\\ \varphi_{P}\left(\sum_{i=1}^{3}E_{i}p_{r_{i}}\right) & = & \varphi_{P}\left(\sum_{i=1}^{3}E_{i}p_{r_{i}}^{\prime }\right)\\ \left(p_{r_{1}},\;p_{r_{2}},\;p_{r_{3}}\right)A_{1} & = & \left(p_{r_{1}}^{\prime },\;p_{r_{2}}^{\prime },\;p_{r_{3}}^{\prime }\right)A_{1} \end{array}$$

where $p_{r_{i}},p_{r_{i}}^{\prime }\in F_{q}^{\beta }$ for 1≤i≤3. This implies that

$$p_{r_{i}}=p_{r_{i}}^{\prime },.$$

Then $p_{r}=p_{r}^{\prime }$. Henceforth, $\varphi_{P}$ is one-one. Take any $\left(p_{r_{1}},\;p_{r_{2}},\;p_{r_{3}}\right)A_{1}\in F_{q}^{3\beta }$; then there exists a corresponding element $p_{r}\in P$ such that $\varphi_{P}\left(p_{r}\right)=\left(p_{r_{1}},\;p_{r_{2}},\;p_{r_{3}}\right)A_{1}$. Therefore, $\varphi_{P}$ is an onto function. Hence, $\varphi_{P}$ is a bijective map.

Moreover, we have

$$\begin{array}{ccc} d_{L}\left(p_{r},\;p_{r}^{\prime }\right) & = & w_{L}\left(p_{r}-p_{r}^{\prime }\right)\\ & = & w_{H}\left(\varphi_{P}\left(p_{r}-p_{r}^{\prime }\right)\right)\\ & = & w_{H}\left(\varphi_{P}\left(p_{r}\right)-\varphi_{P}\left(p_{r}^{\prime }\right)\right)\\ & = & d_{H}\left(\varphi_{P}\left(p_{r}\right),\;\varphi_{P}\left(p_{r}^{\prime }\right)\right). \end{array}$$

Hence, $\varphi_{P}$ is a distance preserving map. □

**Proposition 4.**  *Let $B_{\beta }={⊕}_{i=1}^{3}E_{i}B_{\beta_{i}}$ be a linear code of length β over P. Then*

*(i).*  *
$\varphi_{P}\left(B_{\beta }\right)=B_{\beta_{1}}⊗B_{\beta_{2}}⊗B_{\beta_{3}}.$
*
*(ii).*  *$B_{\beta^{⊥}}={⊕}_{i=1}^{3}E_{i}B_{\beta_{i}^{⊥}}$, further, $B_{\beta }$ is a self-orthogonal code over P if and only if each $B_{\beta_{i}}$ is self-orthogonal code over $F_{q}$ and $B_{\beta }$ is a self-dual code over P if and only if each $B_{\beta_{i}}$ is self-dual code over $F_{q}$, for i=1,2,3.*

**Proof **  1. Let $s=\left(\mu_{0}^{1},\;\mu_{1}^{1},\;\ldots \;,\mu_{\beta -1}^{1},\;\mu_{0}^{2},\;\mu_{1}^{2},\;\ldots \;,\mu_{\beta -1}^{2},\;\mu_{0}^{3},\;\mu_{1}^{3},\;\ldots \;,\mu_{\beta -1}^{3}\right)\in \varphi_{P}\left(B_{\beta }\right)$ and $t_{j}=\sum_{i=1}^{3}\mu_{j}^{i}E_{i}$, for 1≤j≤β−1. So $t=\left(t_{0},\;t_{1},\;\ldots \;,t_{\beta -1}\right)\in B_{\beta }.$ Since $\varphi_{P}$ is a bijective map, $\left(\mu_{0}^{i},\;\mu_{1}^{i},\;\ldots \;,\mu_{\beta -1}^{i}\right)\in B_{\beta_{i}}$ by definition of $B_{\beta_{i}}$ for i=1,2,3, and this implies that $s\in B_{\beta_{1}}⊗B_{\beta_{2}}⊗B_{\beta_{3}}.$ Hence, $\varphi_{P}\left(B_{\beta }\right)\subseteq B_{\beta_{1}}⊗B_{\beta_{2}}⊗B_{\beta_{3}}$.

Conversely, let $s=(\mu_{0}^{1},\;\mu_{1}^{1},\;\ldots \;,\mu_{\beta -1}^{1},\;\mu_{0}^{2},\;\mu_{1}^{2},\;\ldots \;,\mu_{\beta -1}^{2},\;\mu_{0}^{3},\;\mu_{1}^{3},\;\ldots \;,\mu_{\beta -1}^{3}\in B_{\beta_{1}}⊗B_{\beta_{2}}⊗B_{\beta_{3}}$ then $\left(\mu_{0}^{i},\;\mu_{1}^{i},\;\ldots \;,\mu_{\beta -1}^{i}\right)\in B_{\beta_{i}}$ for i=1,2,3. We choose $t_{j}=\sum_{i=1}^{3}\mu_{j}^{i}E_{i}$ for 1≤j≤β−1, then $t=\left(t_{0},\;t_{1},\;\ldots \;,t_{\beta -1}\right)\in B_{\beta }$ and $\varphi_{P}\left(t\right)=s.$ Hence, $s\in \varphi (B_{\beta })$. Moreover, since $\varphi_{P}$ is a bijective map, $|B_{\beta }|=|\varphi_{P}\left(B_{\beta }\right)|.$, then $|B_{\beta }|=|B_{\beta_{1}}⊗B_{\beta_{2}}⊗B_{\beta_{3}}|.$

2. Let $D_{j}=\{t_{j}\in F_{q}^{\beta }\;|\;\sum_{i=1}^{3}E_{i}t_{i}\in B_{\beta }^{⊥}$ for some $t_{j}\in F_{q}^{n}$, i≠j and 1≤i,j≤3}. Then, $B_{\beta }^{⊥}$ is uniquely represented as $B_{\beta }^{⊥}=E_{1}D_{1}⊕E_{2}D_{2}⊕E_{3}D_{3}$. Since $D_{1}$ = {$t_{1}\in F_{q}^{\beta }$ such that $\sum_{i=1}^{3}E_{i}t_{i}\in B_{\beta }^{⊥}$, for some $t_{i}\in F_{q}^{\beta }$, i≠1 and 1≤i≤3}. Clearly, $B_{\beta_{1}}D_{1}=0$, so $D_{1}\subseteq B_{\beta_{1}}^{⊥}.$ Let $E_{1}\in B_{\beta_{1}}^{⊥}$; then $E_{1}x_{1}=0$ for any $c=\sum_{i=1}^{4}E_{i}x_{i}\in B_{\beta }.$ So $E_{1}E_{1}c=E_{1}E_{1}x_{1}=0$ and this implies that $E_{1}E_{1}\in B_{\beta_{1}}^{⊥}$. We have $E_{1}\in D_{1}$ by the unique representation of $B_{\beta }^{⊥}$, so $B_{\beta_{1}}^{⊥}\subseteq D_{1}.$ Similarly, we can show $B_{\beta_{j}}^{⊥}=D_{j}^{⊥}$ for i=1,2,3. Thus $B_{\beta }^{⊥}={⊕}_{i=1}^{3}E_{i}B_{\beta_{i}}^{⊥}$. Moreover, $B_{\beta }$ is a self-orthogonal code over P if and only if $B_{\beta }\subseteq B_{\beta }^{⊥}$. This shows that $E_{1}B_{\beta_{1}}⊕E_{2}B_{\beta_{2}}⊕E_{3}B_{\beta_{3}}\subseteq E_{1}B_{\beta_{1}}^{⊥}⊕B_{\beta_{2}}E_{2}^{⊥}⊕E_{3}B_{\beta_{3}}^{⊥}$, for i=1,2,3. Hence, $B_{\beta }$ is self-orthogonal code over P if and only if each $B_{\beta_{i}}$ is self orthogonal code over $F_{q}$. Similarly, $B_{\beta }$ is self-dual code over P if and only if each $B_{\beta_{i}}$ is self-dual code over $F_{q}$, for i=1,2,3. □

**Proposition 5.**  *Let $B_{\beta }$ be a linear code of length β over P. If $B_{\beta }$ is a self-orthogonal, then $\varphi_{P}\left(B_{\beta }\right)$ is self-orthogonal.*

**Proof.**  Let $p_{r},p_{r}^{\prime }\in B_{\beta }$. Now $p_{r}=\left(p_{r_{0}},p_{r_{1}},\cdots ,p_{r_{\beta -1}}\right),p_{r}^{\prime }=\left(p_{r_{0}}^{\prime },p_{r_{1}}^{\prime },\cdots ,p_{r_{\beta -1}}^{\prime }\right)$, where $p_{j}=\sum_{i=1}^{3}E_{i}p_{r_{i,j}},p_{j}^{\prime }=\sum_{i=1}^{3}E_{i}p_{r_{i,j}}^{\prime },p_{r_{i,j}},p_{r_{i,j}}^{\prime }\in F_{q}$, for i=1,2,3 and j=0,1,2,⋯,β−1. Next, let us consider that $p_{r}\cdot p_{r}^{\prime }=0$. Then, we obtain

$$\begin{array}{ccc} \sum_{i=1}^{\beta -1}p_{j}p_{j}^{\prime } & = & 0\\ \Rightarrow \sum_{j=0}^{\beta -1}\left(\sum_{i=1}^{3}E_{i}p_{r_{i,j}}\right)\left(\sum_{i=1}^{3}E_{i}p_{r_{i,j}}^{\prime }\right) & = & 0. \end{array}$$

Since ${\left(E_{i}\right)}^{2}=E_{i}$, we have

$$\begin{array}{ccc} \sum_{j=0}^{\beta -1}\sum_{i=0}^{3}E_{i}p_{r_{i,j}}p_{r_{i,j}}^{\prime } & = & \sum_{i=0}^{3}\sum_{j=0}^{\beta -1}E_{i}p_{r_{i,j}}p_{r_{i,j}}^{\prime }=0. \end{array}$$

Therefore,

$$\begin{array}{ccc} \sum_{j=0}^{n-1}p_{r_{i,j}}p_{r_{i,j}}^{\prime } & = & 0, \end{array}$$

where i=1,2,3. Also,

$$\begin{array}{ccc} \varphi_{P}\left(p_{r}\right)\varphi_{P}\left(p_{r}^{\prime }\right) & = & \sum_{j=0}^{\beta -1}\sum_{i=0}^{3}p_{r_{i,j}}p_{r_{i,j}}^{\prime }\\ & = & \sum_{i=0}^{3}\sum_{j=0}^{\beta -1}p_{r_{i,j}}p_{r_{i,j}}^{\prime }\\ & = & 0. \end{array}$$

This implies that,

$$\begin{array}{ccc} \varphi_{P}\left(C^{⊥}\right) & \subseteq & \varphi_{P}{\left(C\right)}^{⊥}. \end{array}$$

Since $\varphi_{P}$ is a bijection, then $|\varphi_{P}\left(C^{⊥}\right)|=|\varphi_{P}{\left(C\right)}^{⊥}|$. Hence, $\varphi_{P}\left(C^{⊥}\right)=\varphi_{P}{\left(C\right)}^{⊥}$. Now, *C* is self-orthogonal if and only if $C\subseteq C^{⊥}$. Henceforth, $\varphi_{P}\left(C\right)\subseteq \varphi_{P}\left(C^{⊥}\right)=\varphi_{P}{\left(C\right)}^{⊥}$ if and only if $\varphi_{P}\left(C\right)$ is self-orthogonal. □

An element $q_{r}$ of *Q* is of the form $q_{r}=b_{1}+ub_{2}+vb_{3}+uvb_{4}+v^{2}b_{5}+uv^{2}b_{6}$, for $b_{1},b_{2},b_{3},b_{4},b_{5},b_{6}\in F_{q}$. With the help of Chinese remainder theorem, it is clear to observe that $Q=\zeta_{1}F_{q}⊕\zeta_{2}F_{q}⊕\zeta_{3}F_{q}⊕\zeta_{4}F_{q}⊕\zeta_{5}F_{q}⊕\zeta_{6}F_{q}$. Hence, *Q* is semi-local, commutative, and nonchain ring. Moreover, each $q_{r}$ has a unique representation, $q_{r}=\sum_{j=1}^{6}\delta_{j}b_{j}=\sum_{j=1}^{6}\zeta_{j}q_{r_{j}}$, where $b_{j}$, $q_{r_{j}}$$\in F_{q}$, for 1≤j≤6. We define the Gray map

(2) $$\varphi_{Q}:Q⟶F_{q}^{6}$$

by $\varphi_{Q}\left(q_{r}\right)=\varphi_{Q}\left(\zeta_{1}q_{r_{1}}+\zeta_{2}q_{r_{1}}+\zeta_{3}q_{r_{3}}+\zeta_{4}q_{r_{4}}+\zeta_{5}q_{r_{5}}+\zeta_{6}q_{r_{6}}\right)=\left(q_{r_{1}},q_{r_{2}},q_{r_{3}},q_{r_{4}},q_{r_{5}},q_{r_{6}}\right)A_{2}=sA_{2},$ where $A_{2}\in GL_{6}\left(F_{q}\right)$ is a fixed matrix and $GL_{6}\left(F_{q}\right)$ is the linear group of all 6×6 invertible matrices over the field $F_{q}$ such that $A_{2}A_{2}^{T}\;=\;\lambda I_{6\times 6},\;A_{2}^{T}$ is the transpose of $A_{2}$ and $\lambda \in F_{q}\setminus \left\{0\right\}$ and we use s for the vector $\left(q_{r_{1}},q_{r_{2}},q_{r_{3}},q_{r_{4}},q_{r_{5}},q_{r_{6}}\right)$.

The above-described map (2) can be extended component-wise as

$$\varphi_{Q}:Q^{\beta }⟶F_{q}^{6\gamma }$$

$\left(q_{0},q_{1},\ldots ,q_{\gamma -1}\right)⟶(\left(q_{r_{0,1}},q_{r_{0,2}},q_{r_{0,3}},q_{r_{0,4}},q_{r_{0,5}},q_{r_{0,6}}\right)A_{2},\left(q_{r_{1,1}},q_{r_{1,2}},q_{r_{1,3}},q_{r_{1,4}},q_{r_{1,5}},q_{r_{1,6}}\right)A_{2},\ldots ,\left(q_{r_{\gamma -1,1}},q_{r_{\gamma -1,2}},q_{r_{\gamma -1,3}},q_{r_{\gamma -1,4}},q_{r_{\gamma -1,5}},q_{r_{\gamma -1,6}}\right)$ $A_{2})=s_{0}A_{2},s_{1}A_{2},s_{2}A_{2},\ldots ,s_{\gamma -1}A_{2}$. We write $q_{r}=\left(q_{0},q_{1},\ldots ,q_{\gamma -1}\right)$ and $q_{j}=\zeta_{1}q_{r_{j,1}}+\zeta_{2}q_{r_{j,2}}+\zeta_{3}q_{r_{j,3}}+\zeta_{4}q_{r_{j4}}+\zeta_{5}q_{r_{j,5}}+\zeta_{6}q_{r_{j,6}}\in Q$, where j=0,1,2,…,γ−1. We denote $w_{L}\left(q_{j}\right)=w_{H}\left(\varphi_{Q}\left(q_{j}\right)\right)$ to represent the Lee weight of $q_{j}$, where $w_{H}$ stands for the Hamming weight over $F_{q}$. Let $E_{\gamma }$ be a linear code of length γ over *Q*; we define

$$\begin{array}{ccc} E_{\gamma_{1}} & = & \{q_{r_{1}}\in F_{q}^{\gamma }\;|\;\sum_{j=1}^{6}\zeta_{j}q_{r_{j}}\in E_{\gamma };q_{r_{2}},q_{r_{3}},q_{r_{4}},q_{r_{5}},q_{r_{6}},\in F_{q}^{\gamma },\;1\leq j\leq 6\},\\ E_{\gamma_{2}} & = & \{q_{r_{2}}\in F_{q}^{\gamma }\;|\;\sum_{j=1}^{6}\zeta_{j}q_{r_{j}}\in E_{\gamma };q_{r_{1}},q_{r_{3}},q_{r_{4}},q_{r_{5}},q_{r_{6}},\in F_{q}^{\gamma },\;1\leq j\leq 6\},\\ E_{\gamma_{3}} & = & \{q_{r_{3}}\in F_{q}^{\gamma }\;|\;\sum_{j=1}^{6}\zeta_{j}q_{r_{j}}\in E_{\gamma };q_{r_{1}},q_{r_{2}},q_{r_{4}},q_{r_{5}},q_{r_{6}},\in F_{q}^{\gamma },\;1\leq j\leq 6\},\\ E_{\gamma_{4}} & = & \{q_{r_{4}}\in F_{q}^{\gamma }\;|\;\sum_{j=1}^{6}\zeta_{j}q_{r_{j}}\in E_{\gamma };q_{r_{1}},q_{r_{2}},q_{r_{3}},q_{r_{5}},q_{r_{6}},\in F_{q}^{\gamma },\;1\leq j\leq 6\},\\ E_{\gamma_{5}} & = & \{q_{r_{5}}\in F_{q}^{\gamma }\;|\;\sum_{j=1}^{6}\zeta_{j}q_{r_{j}}\in E_{\gamma };q_{r_{1}},q_{r_{2}},q_{r_{3}},q_{r_{4}},q_{r_{6}},\in F_{q}^{\gamma },\;1\leq j\leq 6\},\\ E_{\gamma_{6}} & = & \{q_{r_{6}}\in F_{q}^{\gamma }\;|\;\sum_{j=1}^{6}\zeta_{j}q_{r_{j}}\in E_{\gamma };q_{r_{1}},q_{r_{2}},q_{r_{3}},q_{r_{4}},q_{r_{5}},\in F_{q}^{\gamma },\;1\leq j\leq 6\}. \end{array}$$

Then, $E_{\gamma_{j}}$ is a linear code of length γ over $F_{q}$, for j=1,2,3,4,5,6.

We come to the following conclusions for *Q* using similar justifications to those used in the case of *P*.

**Proposition 6.**  *The Gray map $\varphi_{Q}$ is a linear and distance preserving map from $\left(Q^{\gamma },\;d_{L}\right)$ to $\left(F_{q}^{6\gamma },\;d_{H}\right)$, where $d_{L}=d_{H}$.*

**Proposition 7.**  *Let $E_{\gamma }={⊕}_{j=1}^{6}\zeta_{j}E_{\gamma_{j}}$ be a linear code of length γ over Q. Then,*

*(i).*  *
$\varphi_{Q}\left(E_{\gamma }\right)=E_{\gamma_{1}}⊗E_{\gamma_{2}}⊗E_{\gamma_{3}}⊗E_{\gamma_{4}}⊗E_{\gamma_{5}}⊗E_{\gamma_{6}}.$
*
*(ii).*  *$E_{\gamma }^{⊥}={⊕}_{j=1}^{6}\zeta_{j}E_{\gamma_{j}}^{⊥}$; further, $E_{\gamma }$ is self-orthogonal if and only if $E_{\gamma_{j}}$ is self-orthogonal, and $E_{\gamma }$ is self-dual if and only if $E_{\gamma_{j}}$ is self-dual, for j=1,2,3,4,5,6.*

**Proposition 8.**  *Let $E_{\gamma }$ be a linear code of length γ over Q. If $E_{\gamma }$ is a self-orthogonal, then $\varphi_{Q}\left(E_{\gamma }\right)$ is self-orthogonal.*

## 4. Gray Image over $F_{q}\mathrm{PQ}$

In the present section, we describe the Gray map over $F_{q}PQ$ and its related results. In $F_{q}PQ$, every element can be written as $\left(a,p_{r},q_{r}\right)=\left(a,E_{1}p_{r_{1}}+E_{2}p_{r_{2}}+E_{3}p_{r_{3}},\zeta_{1}q_{r_{1}}+\zeta_{2}q_{r_{2}}+\zeta_{3}q_{r_{3}}+\zeta_{4}q_{r_{4}}+\zeta_{5}q_{r_{5}}+\zeta_{6}q_{r_{6}}\right)$, where $a,p_{r},q_{r}$ are in $F_{q},P,Q$, respectively. With the help of the above-described Gray maps (1) and (2), we define new Gary map over $F_{q}PQ$ by

(3) $$\varphi :F_{q}PQ⟶F_{q}^{10}$$

such that

$$\begin{array}{ccc} \varphi (a,p_{r},q_{r}) & = & \left(a,\left(p_{r_{1}},p_{r_{2}},p_{r_{3}}\right)A_{1},\left(q_{r_{1}},q_{r_{2}},q_{r_{3}},q_{r_{4}},q_{r_{5}},q_{r_{6}}\right)A_{2}\right)=\left(a,eA_{1},sA_{2}\right). \end{array}$$

Gray map φ is an $F_{q}$-linear and we can easily extend component-wise over $F_{q}^{\alpha }P^{\beta }Q^{\gamma }$ in the following manner:

$$\varphi :F_{q}^{\alpha }\times P^{\beta }\times Q^{\gamma }⟶F_{q}^{\alpha +3\beta +6\gamma }$$

is defined by

$$\begin{array}{ccc} \left(a_{0},a_{1},\ldots ,a_{\alpha -1},p_{0},p_{1},\ldots ,p_{\beta -1},q_{0},q_{1},\ldots ,q_{\gamma -1}\right) & = & (a_{0},a_{1},\ldots ,a_{\alpha -1},\left(p_{r_{0,1}},p_{r_{0,2}},p_{r_{0,3}}\right)A_{1},\\ & \; & \left(p_{r_{1,1}},p_{r_{1,2}},p_{r_{1,3}}\right)A_{1},\ldots ,(p_{r_{\beta -1,1}},p_{r_{\beta -1,2}},\\ & \; & p_{r_{\beta -1,3}})A_{1},(q_{r_{0,1}},q_{r_{0,2}},q_{r_{0,3}},q_{r_{0,4}},q_{r_{0,5}},\\ & \; & q_{r_{0,6}})A_{2},\left(q_{r_{1,1}},q_{r_{1,2}},q_{r_{1,3}},q_{r_{1,4}},q_{r_{1,5}},q_{r_{1,6}}\right)A_{2},\\ & \; & \ldots ,(q_{r_{\gamma -1,1}},q_{r_{\gamma -1,2}},q_{r_{\gamma -1,3}},q_{r_{\gamma -1,4}},q_{r_{\gamma -1,5}},\\ & \; & q_{r_{\gamma -1,6}})A_{2}),\\ & = & (a,e_{0}A_{1},e_{1}A_{1},\ldots ,e_{\beta -1}A_{1},s_{0}A_{2},s_{1}A_{2},\\ & \; & \ldots ,s_{\gamma -1}A_{2}), \end{array}$$

where $a=\left(a_{0},a_{1},\ldots ,a_{\alpha -1}\right)\;\in F_{q}^{\alpha },\;p_{r}=\left(p_{0},p_{1},\ldots ,p_{\beta -1}\right)\;\in P^{\beta }$ and $q_{r}=\left(q_{0},q_{1},\ldots ,q_{\gamma -1}\right)\;\in Q^{\gamma }$. Here, each $p_{i}=E_{1}p_{r_{i},1}+E_{2}p_{r_{i},2}+E_{3}p_{r_{i},3}$, $q_{j}=\zeta_{1}q_{r_{j},1}+\zeta_{2}q_{r_{j},2}+\zeta_{3}q_{r_{j},3}+\zeta_{4}q_{r_{j},4}+\zeta_{5}q_{r_{j},5}+\zeta_{6}q_{r_{j},6}$ are in *P* and *Q* respectively, where i=0,1,…,β−1 and j=0,1,2,…,γ−1. In the same manner as in [20], we define the Lee weight for the element as

$$w_{L}\left(a^{\prime },p_{r}^{\prime },q_{r}^{\prime }\right)=w_{H}\left(a^{\prime }\right)+w_{L}\left(p_{r}^{\prime }\right)+w_{L}\left(q_{r}^{\prime }\right),\;\forall \;\left(a^{\prime },p_{r}^{\prime },q_{r}^{\prime }\right)\in F_{q}^{\alpha }\times P^{\beta }\times Q^{\gamma },$$

where $w_{H}$ represents the Hamming weight and $w_{L}$ represents the Lee weight. Lee distance between the elements $x^{\prime },y^{\prime }\in F_{q}^{\alpha }\times P^{\beta }\times Q^{\gamma }$ is defined as

$$d_{L}\left(x^{\prime },y^{\prime }\right)=w_{L}\left(x^{\prime }-y^{\prime }\right)=w_{H}\left(\varphi \left(x^{\prime },y^{\prime }\right)\right).$$

Next, we give the results on the Gray map over $F_{q}PQ$.

**Proposition 9.**  *Let φ be the above described Gray map. Then*

*(i).*  *φ is an $F_{q}$-linear and distance preserving map from $F_{q}^{\alpha }P^{\beta }Q^{\gamma }$ to $F_{q}^{\alpha +3\beta +6\gamma }$.*
*(ii).*  *If E is a linear code of length (α+β+γ) over $F_{q}PQ$, then Gray image φ(E) of E is also a linear code with the parameters [$\alpha +3\beta +6\gamma ,k,d_{H}$] over $F_{q}$.*

**Proof. **  (i). We take two arbitrary elements $x^{\prime }$ and $y^{\prime }$ of $F_{q}^{\alpha }P^{\beta }Q^{\gamma }$ such that $x^{\prime }=\left(a^{1},p_{r}^{1},q_{r}^{1}\right)$ and $y^{\prime }=\left(a^{2},p_{r}^{2},q_{r}^{2}\right)$. Here,

$$\begin{array}{ccc} a^{1} & = & a_{0}^{1},a_{1}^{1},a_{2}^{1},\ldots ,a_{\alpha -1}^{1},\\ a^{2} & = & a_{0}^{2},a_{1}^{2},a_{2}^{2},\ldots ,a_{\alpha -1}^{2},\\ p_{r}^{1} & = & E_{1}p_{r_{1}}^{1}+E_{2}p_{r_{2}}^{1}+E_{3}p_{r_{3}}^{1},\\ p_{r}^{2} & = & E_{1}p_{r_{1}}^{2}+E_{2}p_{r_{2}}^{2}+E_{3}p_{r_{3}}^{2},\\ q_{r}^{1} & = & \zeta_{1}q_{r_{1}}^{1}+\zeta_{2}q_{r_{2}}^{1}+\zeta_{3}q_{r_{3}}^{1}+\zeta_{4}q_{r_{4}}^{1}+\zeta_{5}q_{r_{5}}^{1}+\zeta_{6}q_{r_{6}}^{1},\\ q_{r}^{2} & = & \zeta_{1}q_{r_{1}}^{2}+\zeta_{2}q_{r_{2}}^{2}+\zeta_{3}q_{r_{3}}^{2}+\zeta_{4}q_{r_{4}}^{2}+\zeta_{5}q_{r_{5}}^{2}+\zeta_{6}q_{r_{6}}^{2}, \end{array}$$

and $a^{1},a^{2},p_{r}^{1},p_{r}^{2}$, and $q_{r}^{1},q_{r}^{2}$ are in $F_{q}^{\alpha },P^{\beta }$, and $Q^{\gamma }$, respectively. Also, we have

$$\begin{array}{ccc} p_{r_{i}}^{1} & = & (p_{r_{i},0}^{1},p_{r_{i},1}^{1},p_{r_{i},2}^{1},\ldots ,p_{r_{i},\beta -1}^{1}),\\ p_{r_{i}}^{2} & = & \left(p_{r_{i},0}^{2},p_{r_{i},1}^{2},p_{r_{i},2}^{2},\ldots ,p_{r_{i},\beta -1}^{2}\right)\in F_{q}^{\beta },\\ q_{r_{j}}^{1} & = & (q_{r_{j},0}^{1},q_{r_{j},1}^{1},q_{r_{j},2}^{1},\ldots ,q_{r_{j},\gamma -1}^{1}),\\ q_{r_{j}}^{2} & = & \left(q_{r_{j},0}^{2},q_{r_{j},1}^{2},q_{r_{j},2}^{2},\ldots ,q_{r_{j},\gamma -1}^{2}\right)\in F_{q}^{\gamma }. \end{array}$$

where 1≤i≤3 and 1≤j≤6. We take

$$\begin{array}{ccc} \varphi (x^{\prime }+y^{\prime }) & = & (a^{1}+a^{2},p_{r_{1}}^{1}+p_{r_{1}}^{2},p_{r_{2}}^{1}+p_{r_{2}}^{2},p_{r_{3}}^{1}+p_{r_{3}}^{2},q_{r_{1}}^{1}+q_{r_{1}}^{2},q_{r_{2}}^{1}+q_{r_{2}}^{2},q_{r_{3}}^{1}+q_{r_{3}}^{2},q_{r_{4}}^{1}+q_{r_{4}}^{2},\\ & \; & q_{r_{5}}^{1}+q_{r_{5}}^{2},q_{r_{6}}^{1}+q_{r_{6}}^{2})\\ & = & \left(a^{1},p_{r_{1}}^{1},p_{r_{2}}^{1},p_{r_{3}}^{1},q_{r_{1}}^{1},q_{r_{2}}^{1},q_{r_{3}}^{1},q_{r_{4}}^{1},q_{r_{5}}^{1},q_{r_{6}}^{1})+(a^{2},p_{r_{1}}^{2},p_{r_{2}}^{2},p_{r_{3}}^{2},q_{r_{1}}^{2},q_{r_{2}}^{2},q_{r_{3}}^{2},q_{r_{4}}^{2},q_{r_{5}}^{2},q_{r_{6}}^{2}\right)\\ & = & \varphi \left(x^{\prime }\right)+\varphi \left(y^{\prime }\right) \end{array}$$

and also

$$\begin{array}{ccc} \varphi (\omega x^{\prime }) & = & \left(\omega a^{1},\omega p_{r_{1}}^{1},\omega p_{r_{2}}^{1},\omega p_{r_{3}}^{1},\omega q_{r_{1}}^{1},\omega q_{r_{2}}^{1},\omega q_{r_{3}}^{1},\omega q_{r_{4}}^{1},\omega q_{r_{5}}^{1},\omega q_{r_{6}}^{1}\right)\\ & = & \omega \varphi (x^{\prime }), \end{array}$$

where $\omega \in F_{q}$. Hence, φ is an $F_{q}$-linear map.

For the remaining part, we will use the fact that φ is a linear map, so we have

$$d_{L}\left(x^{\prime },y^{\prime }\right)=w_{L}\left(x^{\prime }-y^{\prime }\right)=w_{H}\left(x^{\prime }-y^{\prime }\right)=d_{H}\left(x^{\prime },y^{\prime }\right).$$

Therefore, the result is follows.

(ii). This is directly by the definition of Gray map φ. □

In the next step, we define the quasi cyclic code and generalized quasi cyclic code as follows:

**Definition 8.**  *Suppose $\omega \in F_{q}^{mn}$ such that $\omega =(\omega_{1},\omega_{2},\ldots ,\omega_{n})$, where $\omega_{i}\in F_{q}^{m}$ for i=1,2,…,n. Let ξ be the cyclic shift from $F_{q}^{m}$ to $F_{q}^{m}$ and defined as*

$$\xi \left(a_{0},a_{1},\ldots ,a_{m-1}\right)=\left(a_{m-1},a_{0},\ldots ,a_{m-2}\right).$$

*We define another map from $F_{q}^{mn}$ to $F_{q}^{mn}$ such that*

$$\Omega \left(\omega_{1},\omega_{2},\ldots ,\omega_{n}\right)=\left(\xi \left(\omega_{1}\right),\xi \left(\omega_{2}\right),\ldots ,\xi \left(\omega_{n}\right)\right),$$

*where $\omega \in F_{q}^{mn}$ such that $\omega =(\omega_{1},\omega_{2},\ldots ,\omega_{n})$. From here, a code E is known as a quasi-cyclic code of index n if Ω(E)=E.*

**Definition 9.**  *Let $\omega^{\prime }\in F_{q}^{m_{1}}\times F_{q}^{m_{2}}\times F_{q}^{m_{3}}\times \ldots \times F_{q}^{m_{n}}$ such that $\omega^{\prime }=\left(\omega_{1}^{\prime },\omega_{2}^{\prime },\ldots ,\omega_{n}^{\prime }\right)$, where $\omega_{i}^{\prime }\in F_{q}^{m_{i}}$ such that i=1,2,3,…,n. Now, again, let ξ be the cyclic shift from $F_{q}^{m_{i}}$ to $F_{q}^{m_{i}}$ as*

$$\xi :F_{q}^{m_{i}}⟶F_{q}^{m_{i}}$$

*and defined as*

$$\xi \left(a_{0},a_{1},\ldots ,a_{m_{i}-1}\right)=\left(a_{m_{i}-1},a_{0},\ldots ,a_{m_{i}-2}\right).$$

*Next, we define another map as*

$$\Omega_{g}:F_{q}^{m_{1}}\times F_{q}^{m_{2}}\times \ldots \times F_{q}^{m_{n}}⟶F_{q}^{m_{1}}\times F_{q}^{m_{2}}\times \ldots \times F_{q}^{m_{n}}$$

*such that*

$$\Omega_{g}\left(\omega_{1}^{\prime },\omega_{2}^{\prime },\ldots ,\omega_{n}^{\prime }\right)=\left(\xi \left(\omega_{1}^{\prime }\right),\xi \left(\omega_{2}^{\prime }\right),\ldots ,\xi \left(\omega_{n}^{\prime }\right)\right).$$

*A code E is called a generalized quasi-cyclic code if $\Omega_{g}\left(E\right)=E$.*

In view of the above definition, we prove the following result:

**Theorem 1.**  *Let ξ be the cyclic shift over $F_{q}PQ$, and let φ and $\Omega_{g}$ be the mappings described above. Prove that $\varphi_{\xi }=\Omega_{g}\varphi$.*

**Proof.**  Let $c=\left(a_{0},a_{1},\ldots ,a_{\alpha -1},p_{0},p_{1},\ldots ,p_{\beta -1},q_{0},q_{1},\ldots ,q_{\gamma -1}\right)\in F_{q}^{\alpha }P^{\beta }Q^{\gamma }$, where each

$$p_{i}=E_{1}p_{r_{i},1}+E_{2}p_{r_{i},2}+E_{3}p_{r_{i},3}\in P$$

and

$$q_{j}=\zeta_{1}q_{r_{j},1}+\zeta_{2}q_{r_{j},2}+\zeta_{3}q_{r_{j},3}+\zeta_{4}q_{r_{j},4}+\zeta_{5}q_{r_{j},5}+\zeta_{6}q_{r_{j},6}\in Q$$

for i=0,1,2,…,β−1 and j=0,1,2,…,γ−1. Now, we take

$$\begin{array}{ccc} \varphi_{\xi }\left(c\right) & = & \varphi_{\xi }\left(a_{0},a_{1},\ldots ,a_{\alpha -1},p_{0},p_{1},\ldots ,p_{\beta -1},q_{0},q_{1},\ldots ,q_{\gamma -1}\right)\\ & = & \varphi (a_{\alpha -1},a_{0},a_{1},\ldots ,a_{\alpha -2},p_{\beta -1},p_{0},p_{1},\ldots ,p_{\beta -2},q_{\gamma -1},q_{0},q_{1},\ldots ,q_{\gamma -2})\\ & = & (a_{\alpha -1},a_{0},a_{1},\ldots ,a_{\alpha -2},\left(p_{r_{\beta -1,1}},p_{r_{\beta -1,2}},p_{r_{\beta -1,3}}\right)A_{1},\left(p_{r_{0},1},p_{r_{0},2},p_{r_{0},3}\right)A_{1},\left(p_{r_{1},1},p_{r_{1},2},p_{r_{1},3}\right)A_{1},\\ & \; & \ldots ,\left(p_{r_{\beta -2},1},p_{r_{\beta -2,2}},p_{r_{\beta -2,3}}\right)A_{1},\left(q_{r_{\gamma -1,1}},q_{r_{\gamma -1,2}},q_{r_{\gamma -1,3}},q_{r_{\gamma -1,4}},q_{r_{\gamma -1,5}},q_{r_{\gamma -1,6}}\right)A_{2},(q_{r_{0},1},q_{r_{0},2},\\ & \; & q_{r_{0},3},q_{r_{0},4},q_{r_{0},5},q_{r_{0},6})A_{2},\left(q_{r_{1},1},q_{r_{1},2},q_{r_{1},3},q_{r_{1},4},q_{r_{1},5},q_{r_{1},6}\right)A_{2},\ldots ,(q_{r_{\gamma -2,1}},q_{r_{\gamma -2,2}},q_{r_{\gamma -2,3}},\\ & \; & q_{r_{\gamma -2},4},q_{r_{\gamma -2,5}},\ldots ,q_{r_{\gamma -2},6})A_{2}). \end{array}$$

After that, we will obtain

$$\begin{array}{ccc} \Omega_{g}\varphi \left(E\right) & = & \Omega_{g}\varphi \left(a_{0},a_{1},\ldots ,a_{\alpha -1},p_{0},p_{1},\ldots ,p_{\beta -1},q_{0},q_{1},\ldots ,q_{\gamma -1}\right)\\ & = & \Omega (a_{0},a_{1},\ldots ,a_{\alpha -1},\left(p_{r_{0},1},p_{r_{0},2},p_{r_{0},3}\right)A_{1},\left(p_{r_{1},1},p_{r_{1},2},p_{r_{1},3}\right)A_{1},\ldots ,\left(p_{r_{\beta -1},1},p_{r_{\beta -1},2},p_{r_{\beta -1},3}\right)A_{1},\\ & \; & \left(q_{r_{0},1},q_{r_{0},2},q_{r_{0},3},q_{r_{0},4},q_{r_{0},5},q_{r_{0},6}\right)A_{2},\left(q_{r_{1},1},q_{r_{1},2},q_{r_{1},3},q_{r_{1},4},q_{r_{1},5},q_{r_{1},6}\right)A_{2},\ldots ,(q_{r_{\gamma -1},1},q_{r_{\gamma -1},2},\\ & \; & q_{r_{\gamma -1},3},q_{r_{\gamma -1},4},q_{r_{\gamma -1},5},q_{r_{\gamma -1},6})A_{2})\\ & = & (a_{\alpha -1},a_{0},a_{1},\ldots ,a_{\alpha -2},\left(p_{r_{\beta -1},1},p_{r_{\beta -1},2},p_{r_{\beta -1},3}\right)A_{1},\left(p_{r_{0},1},p_{r_{0},2},p_{r_{0},3}\right)A_{1},\left(p_{r_{1},1},p_{r_{1},2},p_{r_{1},3}\right)A_{1},\\ & \; & \ldots ,\left(p_{r_{\beta -2},1},p_{r_{\beta -2},2},p_{r_{\beta -2},3}\right)A_{1},\left(q_{r_{\gamma -1},1},q_{r_{\gamma -1},2},q_{r_{\gamma -1},3},q_{r_{\gamma -1},4},q_{r_{\gamma -1},5},q_{r_{\gamma -1},6}\right)A_{2},(q_{r_{0},1},q_{r_{0},2},\\ & \; & q_{r_{0},3},q_{r_{0},4},q_{r_{0},5},q_{r_{0},6})A_{2},\left(q_{r_{1},1},q_{r_{1},2},q_{r_{1},3},q_{r_{1},4},q_{r_{1},5},q_{r_{1},6}\right)A_{2},\ldots ,(q_{r_{\gamma -2},1},q_{r_{\gamma -2},2},q_{r_{\gamma -2},3},\\ & \; & q_{r_{\gamma -2},4},q_{r_{\gamma -2},5},\ldots ,q_{r_{\gamma -2},6})A_{2}). \end{array}$$

Hence, we conclude that, $\varphi_{\xi }=\Omega_{g}\varphi$. □

In view of Theorem 1, we obtain following result.

**Theorem 2.**  *Let E be a linear code of length (α+β+γ) over $F_{q}PQ$. Then, the Gray image of a $F_{q}PQ$-cyclic code of length (α+β+γ) is a generalized quasi-cyclic code with an index of 10 over $F_{q}$.*

## 5. Main Results

In this section, we describe the structural properties of cyclic codes over $F_{q},P,Q$, and $F_{q}PQ$ as well as obtain quantum error-correcting codes over $F_{q}PQ$.

### 5.1. Cyclic Codes over $F_{q}$

**Theorem 3 **  ([28], Theorem 12.9)**.** *Let A be a cyclic code of length α of over $F_{q}$. Then there exists a unique polynomial $a\left(\kappa \right)\in \frac{F_{q}\left[\kappa \right]}{\langle \kappa^{\alpha }-1\rangle }$ such that $A_{\alpha }=\langle a\left(\kappa \right)\rangle$ and $a\left(\kappa \right)\;|\;\left(\kappa^{\alpha }-1\right)$. Moreover, the dimension of $A_{\alpha }$ is r=α−deg(a) with $\left\{a\left(\kappa \right),\kappa a\left(\kappa \right),\ldots ,\kappa^{r-1}a\left(\kappa \right)\right\}$ as a basis.*

### 5.2. Cyclic Codes over P

**Theorem 4.**  *Let $B_{\beta }={⊕}_{i=1}^{3}E_{i}B_{\beta_{i}}$ be a linear code of length β over P. Then $B_{\beta }$ is a cyclic code of length β over P if and only if each $B_{\beta_{i}}$ is a cyclic code over $F_{q}$, where i=1,2,3.*

**Proof.**  For any $p_{r}=\left(p_{0},p_{1},p_{2},\ldots ,p_{\beta -1}\right)\in B_{\beta }$. We can also have,

$$p_{i}=E_{1}p_{r_{i,1}}+E_{2}p_{r_{i,2}}+E_{3}p_{r_{i,3}},$$

where $p_{r_{i,1}},p_{r_{i,2}},p_{r_{i,3}}\in F_{q}$ and also

$$p_{r_{1}}=\left(p_{r_{1,0}},p_{r_{1,1}},p_{r_{1,2}},\ldots ,p_{r_{1,\beta -1}}\right)\in B_{\beta_{1}},$$

$$p_{r_{2}}=\left(p_{r_{2,0}},p_{r_{2,1}},p_{r_{2,2}},\ldots ,p_{r_{2,\beta -1}}\right)\in B_{\beta_{2}},$$

$$p_{r_{3}}=\left(p_{r_{3,0}},p_{r_{3,1}},p_{r_{3,2}},\ldots ,p_{r_{3,\beta -1}}\right)\in B_{\beta_{2}}.$$

Here, $p_{r_{1}},p_{r_{2}},p_{r_{3}}$ are in $B_{\beta_{1}},B_{\beta_{2}},B_{\beta_{3}}$ respectively. Next, let us consider that $B_{\beta_{1}},B_{\beta_{2}},B_{\beta_{3}}$ are cyclic code over $F_{q}.$ It means that

$$\xi \left(p_{r_{1}}\right)=\left(p_{r_{1,\beta -1}},p_{r_{1,0}},p_{r_{1,1}},p_{r_{1,2}},\ldots ,p_{r_{1,\beta -2}}\right)\in B_{\beta_{1}},$$

$$\xi \left(p_{r_{2}}\right)=\left(p_{r_{2,\beta -1}},p_{r_{2,0}},p_{r_{2,1}},p_{r_{2,2}},\ldots ,p_{r_{2,\beta -2}}\right)\in B_{\beta_{2}},$$

$$\xi \left(p_{r_{3}}\right)=\left(p_{r_{3,\beta -1}},p_{r_{3,0}},p_{r_{3,1}},p_{r_{3,2}},\ldots ,p_{r_{3,\beta -2}}\right)\in B_{\beta_{3}}.$$

Hence, we have

$$\xi \left(p_{i}\right)=E_{1}\xi \left(p_{r_{i,1}}\right)+E_{2}\xi \left(p_{r_{i,2}}\right)+E_{3}\xi \left(p_{r_{i,3}}\right)\in B_{\beta }.$$

This gives

$$E_{1}\xi \left(p_{r_{1}}\right)+E_{2}\xi \left(p_{r_{2}}\right)+E_{3}\xi \left(p_{r_{3}}\right)=\xi \left(p_{r}\right).$$

Thus, we obtain, $\xi \left(p_{r}\right)\in B_{\beta }$. This implies that $B_{\beta }$ is a cyclic code over *P*.

Conversely, we consider that $B_{\beta }$ is a cyclic code over *P*. Suppose

$$p_{r_{i}}=E_{1}p_{r_{i,1}}+E_{2}p_{r_{i,2}}+E_{3}p_{r_{i,3}},$$

where $p_{r_{i,1}},p_{r_{i,2}},p_{r_{i,3}}\in F_{q}$. Then, for any

$$p_{r_{1}}=\left(p_{r_{1,0}},p_{r_{1,1}},p_{r_{1,2}},\ldots ,p_{r_{1,\beta -1}}\right)\in B_{\beta_{1}},$$

$$p_{r_{2}}=\left(p_{r_{2,0}},p_{r_{2,1}},p_{r_{2,2}},\ldots ,p_{r_{2,\beta -1}}\right)\in B_{\beta_{2}},$$

$$p_{r_{3}}=\left(p_{r_{3,0}},p_{r_{3,1}},p_{r_{3,2}},\ldots ,p_{r_{3,\beta -1}}\right)\in B_{\beta_{3}}.$$

Here, $p_{r_{1}},p_{r_{2}},p_{r_{3}}$ are in $B_{\beta_{1}},B_{\beta_{2}},B_{\beta_{3}}$, respectively. Thus, $p_{r}=\left(p_{0},p_{1},\ldots ,p_{\beta -1}\right)\in B_{\beta }$. By the hypothesis, $\xi \left(p_{r}\right)\in B_{\beta }$ because

$$E_{1}\xi \left(p_{r_{1}}\right)+E_{2}\xi \left(p_{r_{2}}\right)+E_{3}\xi \left(p_{r_{3}}\right)=\xi \left(p_{r}\right)$$

Then, we have

$$E_{1}\xi \left(p_{r_{1}}\right)+E_{2}\xi \left(p_{r_{2}}\right)+E_{3}\xi \left(p_{r_{3}}\right)\in B_{\beta }.$$

Therefore,

$$\xi \left(p_{r_{1}}\right)\in B_{\beta ,1},\xi \left(p_{r_{2}}\right)\in B_{\beta ,2},\xi \left(p_{r_{3}}\right)\in B_{\beta ,3}.$$

This shows that $B_{\beta_{1}},B_{\beta_{2}}$ and $B_{\beta_{3}}$ are cyclic codes over $F_{q}$. □

**Corollary 1.**  *Let $B_{\beta }={⊕}_{i=1}^{3}E_{i}B_{\beta_{i}}$ be a cyclic code of length β over P. Then, $B_{\beta }^{⊥}={⊕}_{i=1}^{3}E_{i}B_{\beta_{i}}^{⊥}$ is also a cyclic code of length β over P if and only if $B_{\beta_{i}}^{⊥}$ are cyclic codes of length β over $F_{q}$, for i=1,2,3.*

**Theorem 5.**  *Let $B_{\beta }={⊕}_{i=1}^{3}E_{i}B_{\beta_{i}}$ be a cyclic code of length β over P and $h_{i}\left(\kappa \right)$ be the generator polynomial of the cyclic code $B_{\beta_{i}}$, where i=1,2,3. Then, $B_{\beta }=\langle E_{1}h_{1}\left(\kappa \right),E_{2}h_{2}\left(\kappa \right),E_{3}h_{3}\left(\kappa \right)\rangle$ and $|B_{\beta }|=q^{3\beta -\sum_{i=1}^{3}deg\left(h_{i}\right)}$.*

**Proof.**  Let $B_{\beta }$ be a cyclic code of length β over *P*. Then, by Theorem 4,

$$B_{\beta_{1}}=\langle h_{1}\left(\kappa \right)\rangle \subseteq \frac{F_{q}\left[\kappa \right]}{\langle \kappa^{\beta }-1\rangle },$$

$$B_{\beta_{2}}=\langle h_{2}\left(\kappa \right)\rangle \subseteq \frac{F_{q}\left[\kappa \right]}{\langle \kappa^{\beta }-1\rangle },$$

$$B_{\beta_{3}}=\langle h_{3}\left(\kappa \right)\rangle \subseteq \frac{F_{q}\left[\kappa \right]}{\langle \kappa^{\beta }-1\rangle },$$

and also

$$B_{\beta }=E_{1}B_{\beta_{1}}⊕E_{2}B_{\beta_{2}}⊕E_{3}B_{\beta_{3}},$$

where

$$h_{1}\left(\kappa \right)\in B_{\beta_{1}},h_{2}\left(\kappa \right)\in B_{\beta_{2}},h_{3}\left(\kappa \right)\in B_{\beta_{3}}.$$

Therefore,

$$\begin{array}{ccc} B_{\beta } & \subseteq & \langle E_{1}h_{1}\left(\kappa \right),E_{2}h_{2}\left(\kappa \right),E_{3}h_{3}\left(\kappa \right)\rangle \\ & \subseteq & \frac{P[\kappa ]}{\langle \kappa^{\beta }-1\rangle }. \end{array}$$

We take any

$$\begin{array}{ccc} E_{1}k_{r_{1}}\left(\kappa \right)h_{1}\left(\kappa \right)+E_{2}k_{r_{2}}\left(\kappa \right)h_{2}\left(\kappa \right)+E_{3}k_{r_{3}}\left(\kappa \right)h_{3}\left(\kappa \right) & \in & \langle E_{1}h_{1}\left(\kappa \right),E_{2}h_{2}\left(\kappa \right),E_{3}h_{3}\left(\kappa \right)\rangle \\ & \subseteq & \frac{P[\kappa ]}{\langle \kappa^{\beta }-1\rangle }. \end{array}$$

Here, $k_{r_{1}}\left(\kappa \right),k_{r_{2}}\left(\kappa \right),k_{r_{3}}\left(\kappa \right)\in \frac{P[\kappa ]}{\langle \kappa^{\beta }-1\rangle }$ and also $h_{1}\left(\kappa \right),h_{2}\left(\kappa \right),h_{3}\left(\kappa \right)\in F_{q}\left[\kappa \right]$ such that

$$\begin{array}{ccc} E_{1}k_{r_{1}}\left(\kappa \right) & = & E_{1}h_{1}\left(\kappa \right),\\ E_{2}k_{r_{2}}\left(\kappa \right) & = & E_{2}h_{2}\left(\kappa \right),\\ E_{3}k_{r_{3}}\left(\kappa \right) & = & E_{3}h_{3}\left(\kappa \right). \end{array}$$

This means that $\langle E_{1}h_{1}\left(\kappa \right),E_{2}h_{2}\left(\kappa \right),E_{3}h_{3}\left(\kappa \right)\rangle \subseteq B_{\beta }$. From the above discussion, we conclude that $\langle E_{1}h_{1}\left(\kappa \right),E_{2}h_{2}\left(\kappa \right),E_{3}h_{3}\left(\kappa \right)\rangle =B_{\beta }$. But,

$$B_{\beta }=|B_{\beta_{1}}\left|\right|B_{\beta_{2}}\left|\right|B_{\beta_{3}}|.$$

This yields that

$$\begin{array}{ccc} |B_{\beta }| & = & q^{\beta -deg(h_{1}\left(\kappa \right))}q^{\beta -deg(h_{2}\left(\kappa \right))}q^{\beta -deg(h_{3}\left(\kappa \right))}\\ & = & q^{3\beta -(deg\left(h_{1}\left(\kappa \right)\right)+deg\left(h_{2}\left(\kappa \right)\right)+deg\left(h_{3}\left(\kappa \right)\right))}\\ |B_{\beta }| & = & q^{3\beta -\sum_{i=1}^{3}deg\left(h_{i}\right)}. \end{array}$$

□

**Theorem 6.**  *Let $B_{\beta }={⊕}_{i=1}^{3}E_{i}B_{\beta_{i}}$ be a cyclic code of length β over P and $h_{i}\left(\kappa \right)$ be the generator polynomial of the cyclic code $B_{\beta_{i}}$, where i=1,2,3. Suppose there exists a unique polynomial h(κ)∈P(κ) such that $B_{\beta }=\langle h\left(\kappa \right)\rangle$ and h(κ) divides $\kappa^{\beta }-1$ and also $h\left(\kappa \right)=E_{1}h_{1}\left(\kappa \right)+E_{2}h_{2}\left(\kappa \right)+E_{3}h_{3}\left(\kappa \right)$.*

**Proof.**  In view of Theorem 5, let $C=\langle E_{1}h_{1}\left(\kappa \right),E_{2}h_{2}\left(\kappa \right),E_{3}h_{3}\left(\kappa \right)\rangle$ and $h_{1}\left(\kappa \right),h_{2}\left(\kappa \right),h_{3}\left(\kappa \right)$ be the monic generator polynomials of $B_{\beta_{1}},B_{\beta_{2}},B_{\beta_{3}}$, respectively. Next, let us consider that $h\left(\kappa \right)=E_{1}h_{1}\left(\kappa \right)+E_{2}h_{2}\left(\kappa \right)+E_{3}h_{3}\left(\kappa \right)$. Obviously, $\langle h\left(\kappa \right)\rangle \subseteq B_{\beta }$. Now,

$$\begin{array}{ccc} E_{1}h_{1}\left(\kappa \right) & = & E_{1}h\left(\kappa \right),\\ E_{2}h_{2}\left(\kappa \right) & = & E_{2}h\left(\kappa \right),\\ E_{3}h_{3}\left(\kappa \right) & = & E_{3}h\left(\kappa \right). \end{array}$$

Also, it means that $B_{\beta }\subseteq \langle h\left(\kappa \right)\rangle$. This is clear from above discussion that $B_{\beta }=\langle h\left(\kappa \right)\rangle$. But $h_{1}\left(\kappa \right),h_{2}\left(\kappa \right),h_{3}\left(\kappa \right)$ are the monic divisor of $\left(\kappa^{\beta }-1\right)$. There are $t_{r_{1}}\left(\kappa \right),t_{r_{2}}\left(\kappa \right),t_{r_{3}}\left(\kappa \right)\in \frac{F_{q}\left[\kappa \right]}{\langle \kappa^{\beta }-1\rangle }$. This implies that

$$\left[E_{1}t_{r_{1}}\left(\kappa \right)+E_{2}t_{r_{2}}\left(\kappa \right)+E_{3}t_{r_{3}}\left(\kappa \right)\right]h\left(\kappa \right)=\kappa^{\beta }-1.$$

Therefore, $h\left(\kappa \right)/\left(\kappa^{\beta }-1\right)$. Hence, h(κ) is unique by the uniqueness of $h_{1}\left(\kappa \right),h_{2}\left(\kappa \right),h_{3}\left(\kappa \right)$. □

**Corollary 2.**  Let $B_{\beta }={⊕}_{i=1}^{3}E_{i}B_{\beta_{i}}$ be a cyclic code of length β over P, $h_{i}\left(\kappa \right)$ be the generator polynomial of the cyclic code $B_{\beta_{i}}$ and $e_{i}^{*}\left(\kappa \right)$ is the reciprocal polynomials of $e_{i}\left(\kappa \right)$ such that $\kappa^{\beta }-1=e_{i}\left(\kappa \right)h_{i}\left(\kappa \right)$ for i=1,2,3. Then, $B_{\beta }^{⊥}=\langle E_{1}e_{1}^{*}\left(\kappa \right),E_{2}e_{2}^{*}\left(\kappa \right),E_{3}e_{3}^{*}\left(\kappa \right)\rangle$ and $|B_{\beta }^{⊥}|=q^{\sum_{i=1}^{3}deg\left(h_{i}\right)}$.

### 5.3. Cyclic Codes over Q

We arrive at the following conclusions for cyclic codes over *Q* using similar justifications to those used in the case of cyclic codes over *P*.

**Theorem 7.**  *Let $E_{\gamma }={⊕}_{j=1}^{6}\zeta_{j}E_{\gamma_{j}}$ be a linear code of length γ over Q. Then $E_{\gamma }$ is a cyclic code of length γ over Q if and only if each $E_{\gamma_{j}}$ is a cyclic code over $F_{q}$, where j=1,2,3,4,5,6.*

**Corollary 3.**  *Let $E_{\gamma }={⊕}_{j=1}^{6}\zeta_{j}E_{\gamma_{j}}$ be a cyclic code of length γ over Q. Then, $E_{\gamma }^{⊥}={⊕}_{j=1}^{6}\zeta_{j}(E_{\gamma_{j}}^{⊥}$ is also a cyclic code of length γ over Q if and only if each $E_{\gamma_{j}}^{⊥}$ is a cyclic code of length γ over $F_{q}$, for i=1,2,3,4,5,6.*

**Theorem 8.**  *Let $E_{\gamma }={⊕}_{j=1}^{6}\zeta_{j}E_{\gamma_{j}}$ be a cyclic code of length γ over Q and $\ell_{j}\left(\kappa \right)$ be the generator polynomial of the cyclic code $E_{\gamma_{j}}$, where j=1,2,3,4,5,6. Then, $E_{\gamma }=\langle \ell \left(\kappa \right)\rangle$ and $|E_{\gamma }|=q^{3\gamma -\sum_{j=1}^{6}deg\left(\ell_{j}\right)}$, where $\ell \left(\kappa \right)=\zeta_{1}\ell_{1}\left(\kappa \right)+\zeta_{2}\ell_{2}\left(\kappa \right)+\zeta_{3}\ell_{3}\left(\kappa \right)+\zeta_{4}\ell_{4}\left(\kappa \right)+\zeta_{5}\ell_{5}\left(\kappa \right)+\zeta_{6}\ell_{6}\left(\kappa \right)$.*

**Theorem 9.**  *Let $E_{\gamma }={⊕}_{j=1}^{6}\zeta_{j}E_{\gamma_{j}}$ be a cyclic code of length γ over Q and $\ell_{j}\left(\kappa \right)$ be the generator polynomial of the cyclic code $E_{\gamma_{j}}$, where j=1,2,3,4,5,6. Suppose there exists a unique polynomial ℓ(κ)∈Q(κ) such that $E_{\gamma }=\langle \ell \left(\kappa \right)\rangle$ and ℓ(κ) divides $\kappa^{\gamma }-1$ and also $\ell \left(\kappa \right)=\zeta_{1}\ell_{1}\left(\kappa \right)+\zeta_{2}\ell_{2}\left(\kappa \right)+\zeta_{3}\ell_{3}\left(\kappa \right)+\zeta_{4}\ell_{4}\left(\kappa \right)+\zeta_{5}\ell_{5}\left(\kappa \right)+\zeta_{6}\ell_{6}\left(\kappa \right)$.*

**Corollary 4.**  Let $E_{\gamma }={⊕}_{j=1}^{6}\zeta_{j}E_{\gamma_{j}}$ be a cyclic code of length γ over Q. Suppose $\ell_{j}\left(\kappa \right)$ is the generator polynomial of the cyclic code $E_{\gamma_{j}}$ and $k_{j}^{*}\left(\kappa \right)$ is the reciprocal polynomials of $k_{j}\left(\kappa \right)$ such that $\kappa^{\gamma }-1=k_{j}\left(\kappa \right)\ell_{j}\left(\kappa \right)$ for j=1,2,3,4,5,6. Then, $E_{\gamma }^{⊥}=\langle \zeta_{1}k_{1}^{*}\left(\kappa \right),\zeta_{2}k_{2}^{*}\left(\kappa \right),\zeta_{3}k_{3}^{*}\left(\kappa \right),\zeta_{4}k_{4}^{*}\left(\kappa \right),\zeta_{5}k_{5}^{*}\left(\kappa \right),\zeta_{6}k_{6}^{*}\left(\kappa \right),\rangle$ and $|(E_{\gamma }^{⊥}|=q^{\sum_{j=1}^{6}deg\left(\ell_{j}^{*}\right)}$.

### 5.4. Cyclic Codes over $F_{q}PQ$

In the present section, we discuss the generator polynomial of E over $F_{q}PQ$. We begin with the following result:

**Theorem 10.**  *Let E be a cyclic code over $F_{q}PQ$. Then,*

$$E=\langle (a\left(\kappa \right)|0|0),(0|h\left(\kappa \right)|0),(r_{1}\left(\kappa \right)|r_{2}\left(\kappa \right)|\ell \left(\kappa \right))\rangle ,$$

*where $a\left(\kappa \right)|\left(\kappa^{\alpha }-1\right),h\left(\kappa \right)\left|\left(\kappa^{\beta }-1\right),\ell \left(\kappa \right)\right|\left(\kappa^{\gamma }-1\right)$ and also $r_{1}\left(\kappa \right)\in \frac{F_{q}\left[\kappa \right]}{\langle \kappa^{\alpha }-1\rangle }$, $r_{2}\left(\kappa \right)\in \frac{F_{q}\left[\kappa \right]}{\langle \kappa^{\beta }-1\rangle }$.*

**Proof.**  In view of Theorems 3, 5 and 8, we define

$$\begin{array}{ccc} A_{\alpha } & = & \langle a(\kappa )\rangle ,\\ B_{\beta } & = & \langle h(\kappa )\rangle ,\\ E_{\gamma } & = & \langle \ell (\kappa )\rangle , \end{array}$$

where $a\left(\kappa \right)|\left(\kappa^{\alpha }-1\right),\;h\left(\kappa \right)\left|\left(\kappa^{\beta }-1\right),\;\ell \left(\kappa \right)\right|\left(\kappa^{\gamma }-1\right)$. Then, the proof is similar as in [20]. □

**Definition 10**  ([25])**.** *A $F_{q}PQ$-linear code E of length (α+β+γ) is called a separable code if $E=A_{\alpha }^{\prime }⊗B_{\beta }^{\prime }⊗E_{\gamma }^{\prime }$ while considering $A_{\alpha }^{\prime }$, $B_{\beta }^{\prime }$ and $E_{\gamma }^{\prime }$ as punctured code of E by deleting the coordinate outside the α,β and γ components, respectively.*

**Lemma 1.**  *Let $E=\langle (a\left(\kappa \right)|0|0),(0|h\left(\kappa \right)|0),(r_{1}\left(\kappa \right)|r_{2}\left(\kappa \right)|\ell \left(\kappa \right))\rangle$ be a $F_{q}PQ$-cyclic code. Then,*

*(i)*  *$deg\left(r_{1}\left(\kappa \right)\right)\leq deg\left(a\left(\kappa \right)\right)$, $deg\left(r_{2}\left(\kappa \right)\right)\leq deg\left(h\left(\kappa \right)\right)$ and $a\left(\kappa \right)|h\left(\kappa \right)r_{1}\left(\kappa \right)$, $h\left(\kappa \right)|\ell \left(\kappa \right)r_{2}\left(\kappa \right)$.*
*(ii)*  *$A_{\alpha }^{\prime }=\langle gcd\left(a\left(\kappa \right),r_{1}\left(\kappa \right)\right)\rangle$, $B_{\beta }^{\prime }=\langle gcd\left(h\left(\kappa \right),r_{2}\left(\kappa \right)\right)\rangle$ and $E_{\gamma }^{\prime }=\langle \ell \left(\kappa \right)\rangle$.*

**Proof.**  The proof is parallel to that of Lemmas 3.2, 3.3 and 3.4 of [20]. □

**Lemma 2.**  *Let $E=\langle (a\left(\kappa \right)|0|0),(0|h\left(\kappa \right)|0),(r_{1}\left(\kappa \right)|r_{2}\left(\kappa \right)|\ell \left(\kappa \right))\rangle$ be a $F_{q}PQ$-cyclic code. Then,*

*(i)*  *$a\left(\kappa \right)|r_{1}\left(\kappa \right)$ if and only if $r_{1}\left(\kappa \right)=0$.*
*(ii)*  *$h\left(\kappa \right)|r_{2}\left(\kappa \right)$ if and only if $r_{2}\left(\kappa \right)=0$.*

**Proof.**  Proof is parallel to that of Lemmas 5.8 and 5.9 of [20]. □

The following Lemma is a direct consequence of Lemma 2.

**Lemma 3.**  *Let $E=\langle (a\left(\kappa \right)|0|0),(0|h\left(\kappa \right)|0),(r_{1}\left(\kappa \right)|r_{2}\left(\kappa \right)|\ell \left(\kappa \right))\rangle$ be a $F_{q}PQ$-cyclic code. Then, the following are equivalent:*

*(i)*  *E is separable.*
*(ii)*  *$a\left(\kappa \right)|r_{1}\left(\kappa \right)$, $h\left(\kappa \right)|r_{2}\left(\kappa \right)$.*
*(iii)*  *E=〈(a(κ)∣0∣0),(0∣h(κ)∣0),(0∣0∣ℓ(κ))〉.*

*Consequently, for a separable code, we have*

$$\begin{array}{ccc} A_{\alpha }^{\prime } & = & \langle gcd\left(a\left(\kappa \right),r_{1}\left(\kappa \right)\right)\rangle =\langle a\left(\kappa \right)\rangle =A_{\alpha },\\ B_{\beta }^{\prime } & = & \langle gcd\left(h\left(\kappa \right),r_{2}\left(\kappa \right)\right)\rangle =\langle h\left(\kappa \right)\rangle =B_{\beta },\\ E_{\gamma }^{\prime } & = & \langle \ell \left(\kappa \right)\rangle =E_{\gamma }. \end{array}$$

**Theorem 11.**  *Let $E=A_{\alpha }⊗B_{\beta }⊗E_{\gamma }$ be a $F_{q}PQ$-linear code of length (α+β+γ), where $A_{\alpha },B_{\beta }$ and $E_{\gamma }$ are linear code of α,β and γ, respectively. Then, E is a $F_{q}PQ$ cyclic code of length (α+β+γ) if and only if $A_{\alpha },B_{\beta }$ and $E_{\gamma }$ are cyclic codes of length α,β and γ over $F_{q},P$ and Q, respectively.*

**Proof.**  First, we suppose that E is a $F_{q}PQ$-cyclic code of length (α+β+γ) and c∈E, where

$$c=(a_{0},a_{1},\ldots ,a_{\alpha -1},p_{0},p_{1},\ldots ,p_{\beta -1},q_{0},q_{1},\ldots ,q_{\gamma -1})$$

and also

$$\begin{array}{cc} \left(a_{0},a_{1},\ldots ,a_{\alpha -1}\right) & \in A_{\alpha },\\ \left(p_{0},p_{1},\ldots ,p_{\beta -1}\right) & \in B_{\beta },\\ \left(q_{0},q_{1},\ldots ,q_{\gamma -1}\right) & \in E_{\gamma }. \end{array}$$

By the definition of cyclic code, we have

$$(a_{\alpha -1},a_{0},a_{1},\ldots ,a_{\alpha -2},p_{\beta -1}p_{0},p_{1},\ldots ,p_{\beta -2},q_{\gamma -1},q_{0},q_{1},\ldots ,q_{\gamma -2})\in E$$

Now,

$$\begin{array}{cc} \left(a_{\alpha -1},a_{0},a_{1},\ldots ,a_{\alpha -2}\right) & \in A_{\alpha },\\ \left(p_{\beta -1},p_{0},p_{1},\ldots ,p_{\beta -2}\right) & \in B_{\beta },\\ \left(q_{\gamma -1},q_{0},q_{1},\ldots ,q_{\gamma -2}\right) & \in E_{\gamma }. \end{array}$$

Hence, $A_{\alpha },B_{\beta }$ and $E_{\gamma }$ are cyclic codes of length α,β and γ over $F_{q},P$ and *Q*, respectively.

For the converse part, we consider that $A_{\alpha },B_{\beta }$ and $E_{\gamma }$ are cyclic codes of length α,β and γ over $F_{q},P$ and *Q*, respectively, and next, we will prove that $E=A_{\alpha }⊗B_{\beta }⊗E_{\gamma }$ is a cyclic code over $F_{q}PQ$. Hence,

$$\begin{array}{cc} \left(a_{0},a_{1},\ldots ,a_{\alpha -1}\right) & \in A_{\alpha },\\ \left(p_{0},p_{1},\ldots ,p_{\beta -1}\right) & \in B_{\beta },\\ \left(q_{0},q_{1},\ldots ,q_{\gamma -1}\right) & \in E_{\gamma }. \end{array}$$

But, all are cyclic, and we have

$$\begin{array}{cc} \left(a_{\alpha -1},a_{0},a_{1},\ldots ,a_{\alpha -2}\right) & \in A_{\alpha },\\ \left(p_{\beta -1},p_{0},p_{1},\ldots ,p_{\beta -2}\right) & \in B_{\beta },\\ \left(q_{\gamma -1},q_{0},q_{1},\ldots ,q_{\gamma -2}\right) & \in E_{\gamma }. \end{array}$$

Thus,

$$\left(a_{\alpha -1},a_{0},a_{1},\ldots ,a_{\alpha -2},p_{\beta -1},p_{0},p_{1},\ldots ,p_{\beta -2},q_{\gamma -1},q_{0},q_{1},\ldots ,q_{\gamma -2}\right)\in A_{\alpha }⊗B_{\beta }⊗E_{\gamma }=E.$$

Consequently, E is a $F_{q}PQ$-cyclic code of length (α+β+γ). □

In view of Theorems 4, 7 and 11, we have the following result:

**Corollary 5.**  *Let $E=A_{\alpha }⊗B_{\beta }⊗E_{\gamma }$ be a $F_{q}PQ$-linear code of length (α+β+γ) such that $A_{\alpha },B_{\beta }$ and $E_{\gamma }$ are the linear codes of length α,β and γ over $F_{q}$, P and Q, respectively. Then, E is a $F_{q}PQ$-cyclic code of length (α+β+γ) if and only if $A_{\alpha },B_{\beta_{i}}$ and $E_{\gamma_{j}}$ are the cyclic codes of length α,β and γ over $F_{q}$, P and Q for i=1,2,3 and j=1,2,3,4,5,6.*

In Theorem 10, we studied the generator polynomial for a $F_{q}PQ$-cyclic code of length (α+β+γ). Here, we examine the generator polynomial for a separable $F_{q}PQ$-cyclic code of length (α+β+γ) in the manner described below.

**Theorem 12.**  *Let $E=A_{\alpha }⊗B_{\beta }⊗E_{\gamma }$ be a $F_{q}PQ$-cyclic code of length (α+β+γ), where $A_{\alpha }=\langle a\left(\kappa \right)\rangle$, $B_{\beta }=\langle h\left(\kappa \right)\rangle$ and $E_{\gamma }=\langle \ell \left(\kappa \right)\rangle$. Then, C=〈a(κ)〉⊗〈h(κ)〉⊗〈ℓ(κ)〉.*

**Proof.**  We have

$$\begin{array}{ccc} A_{\alpha } & = & \langle a(\kappa )\rangle ,\\ B_{\beta } & = & \langle h(\kappa )\rangle ,\\ E_{\gamma } & = & \langle \ell (\kappa )\rangle . \end{array}$$

Then, the proof directly follows. □

**Example 1.**  *Let α=11,β=7,γ=8 and $Q_{11+7+8}=\frac{F_{27}\left[\kappa \right]}{\langle \kappa^{11}-1\rangle }\times \frac{P[\kappa ]}{\langle \kappa^{7}-1\rangle }\times \frac{Q[\kappa ]}{\langle \kappa^{8}-1\rangle }$, where $P=F_{27}\left[v\right]/\langle v^{3}-\alpha_{2}^{2}v\rangle$ and $Q=F_{27}\left[u,v\right]/\langle u^{2}-\alpha_{1}^{2},v^{3}-\alpha_{2}^{2}v\rangle$. We take $F_{27}=\frac{F_{3}}{\langle \kappa^{3}+2\kappa +1\rangle }$. It can be easily seen that $\kappa^{3}+2\kappa +1$ is a irreducible in $F_{3}$ and ω be a zero of polynomial in $F_{27}$, then,*

$$\kappa^{11}-1=\left(\kappa +2\right)\left(\kappa^{5}+2\kappa^{3}+\kappa^{2}+2\kappa +2\right)\left(\kappa^{5}+\kappa^{4}+2\kappa^{3}+\kappa^{2}+2\right)\in F_{27}\left[\kappa \right].$$

*Let $a\left(\kappa \right)=\left(\kappa +2\right)\left(\kappa^{5}+2\kappa^{3}+\kappa^{2}+2\kappa +2\right)$. Then, $A_{\alpha }=\langle a\left(\kappa \right)\rangle$ is a cyclic code of length 11 over $F_{27}$. Also, we have,*

$$\kappa^{7}-1=\left(\kappa +2\right)\left(\kappa^{2}+\omega^{5}\kappa +1\right)\left(\kappa^{2}+\omega^{15}\kappa +1\right)\left(\kappa^{2}+\omega^{19}\kappa +1\right)\in F_{27}\left[\kappa \right].$$

*Let $h_{1}\left(\kappa \right)=\left(\kappa +2\right)\left(\kappa^{2}+\omega^{5}\kappa +1\right),h_{2}\left(\kappa \right)=\left(\kappa +2\right)\left(\kappa^{2}+\omega^{15}\kappa +1\right)$ and $h_{3}\left(\kappa \right)=\left(\kappa +2\right)\left(\kappa^{2}+\omega^{19}\kappa +1\right)$. Thus $B_{\beta ,i}=\langle h_{i}\left(\kappa \right)\rangle$ are cyclic codes of length 7 over $F_{27}$, for i=1,2,3. Therefore, $B_{\beta }=\langle h\left(\kappa \right)\rangle$ is a cyclic code of length 7 over P.*

*Now, we have*

$$\kappa^{8}-1=\left(\kappa +1\right)\left(\kappa +2\right)\left(\kappa^{2}+\kappa +2\right)\left(\kappa^{2}+2\kappa +2\right)\left(\kappa^{2}+1\right)\in F_{27}\left[\kappa \right].$$

*Let $\ell_{1}\left(\kappa \right)=\ell_{2}\left(\kappa \right)=\left(\kappa +1\right)\left(\kappa +2\right)\left(\kappa^{2}+\kappa +2\right)$, $\ell_{3}\left(\kappa \right)=\ell_{4}\left(\kappa \right)=\left(\kappa +1\right)\left(\kappa +2\right)\left(\kappa^{2}+2\kappa +2\right)$ and $\ell_{5}\left(\kappa \right)=\ell_{6}\left(\kappa \right)=\left(\kappa +1\right)\left(\kappa +2\right)\left(\kappa^{2}+1\right)$. $E_{\gamma ,j}=\langle \ell_{j}\rangle$ are cyclic codes of length 8 over $F_{27}$, where j=1,2,3,4,5,6. Thus, $E_{\gamma }=\langle \ell \left(\kappa \right)\rangle$ is a cyclic code of length 8 over Q, where $\ell \left(\kappa \right)=\zeta_{1}\ell_{1}\left(\kappa \right)+\zeta_{2}\ell_{2}\left(\kappa \right)+\zeta_{3}\ell_{3}\left(\kappa \right)+\zeta_{4}\ell_{4}\left(\kappa \right)+\zeta_{5}\ell_{5}\left(\kappa \right)+\zeta_{6}\ell_{6}\left(\kappa \right)$. Hence, $E=\langle (a\left(\kappa \right)|0|0),(0|h\left(\kappa \right)|0),(r_{1}\left(\kappa \right)|r_{2}\left(\kappa \right)|\ell \left(\kappa \right))\rangle$ = 〈a(κ)〉⊗〈h(κ)〉⊗〈ℓ(κ)〉 is a separable $F_{q}PQ$-cyclic code of length (11+7+8).*

### 5.5. Quantum Error-Correcting Codes

In the present section, we will explore how to obtain quantum codes using the Calderbank–Shor–Steane (CSS) construction from [29], which utilizes dual-containing cyclic codes. The CSS construction is a powerful method for constructing quantum codes with desirable properties. By employing this construction, we can create quantum codes that outperform existing codes in terms of their parameters, such as dimension and minimum distance. We use a necessary and sufficient condition over the finite fields to obtain the condition for cyclic codes to contain their duals. It must be stated that the set of *n*-fold tensor product ${\left(C^{q}\right)}^{⊗n}=C^{q}⊗C^{q}⊗\ldots ⊗C^{q}$ (*n*-times) is a Hilbert space of dimension $q^{n}$, and also $C^{q}$ is the Hilbert space of dimension *q*, where C is the complex field. A quantum code is the subspace of Hilbert space ${\left(C^{q}\right)}^{⊗n}$. A quantum code of length *n* over the field $F_{q}$ (*q* is a power of a prime.) is denoted by ${\left[\left[n,k,d\right]\right]}_{q}$, where *k* is the dimension, and *d* is the minimum distance. We know that each quantum code satisfies the singleton bound, i.e., n−k+2≥2d. A quantum code is said to be MDS (maximum distance separable) if n−k+2=2d.

To construct better quantum codes compared to existing ones, we focus on two main conditions:

**Higher Dimension (k):** One way to improve a quantum code is by increasing its dimension, denoted as *k*. The dimension represents the number of encoded qubits or logical operators that can be stored in the code. By constructing a CSS code with a higher dimension compared to existing codes, we can encode more information in the same number of physical qubits, leading to increased storage capacity and computational capabilities.

**Larger Minimum Distance (d):** The minimum distance, denoted as *d*, of a quantum code determines its error-correcting capability. A larger minimum distance implies better error detection and correction properties. By constructing a CSS code with a larger minimum distance compared to existing codes, we enhance its ability to protect against errors and improve the overall reliability of the encoded information.

A quantum code ${\left[\left[n,k,d\right]\right]}_{q}$ is better than the other quantum code ${\left[\left[n^{\prime },k^{\prime },d^{\prime }\right]\right]}_{q}$ if one or both the following conditions hold:

1. $\frac{k}{n}>\frac{k^{\prime }}{n^{\prime }}$, where $d=d^{\prime }$(larger code rate with same distance);
2. $d>d^{\prime }$ where $\frac{k}{n}=\frac{k^{\prime }}{n^{\prime }}$ (larger distance with the same code rate).

In summary, the CSS construction, utilizing dual-containing cyclic codes, allows us to construct quantum codes. By carefully selecting the parameters of the codes involved, we can create better quantum codes compared to existing ones, with improved dimensions and minimum distances.

**Lemma 4**  ([29])**.** *[CSS Construction] If E is an [n, k, d] linear code with $(E^{⊥}\subseteq C$ over $F_{q}$, then there exists a QEC code with parameters ${\left[\left[n,2k-n,d\right]\right]}_{q}$ over $F_{q}$.*

**Lemma 5**  ([30])**.** *A cyclic code E of length n over $F_{q}$ with generator polynomial f(κ) that contains its dual if and only if*

$$\kappa^{n}-1\;\equiv \;0\;mod\left(f\left(\kappa \right)f^{*}\left(\kappa \right)\right),$$

where $f^{*}\left(\kappa \right)$ is the reciprocal polynomial of f(κ).

**Proposition 10.**  *Let E be a $F_{q}PQ$-linear code of length (α+β+γ). Then, the Gray image of E, i.e., $\varphi \left(E\right)=A_{\alpha }⊗B_{\beta_{1}}⊗B_{\beta_{2}}⊗B_{\beta_{3}}⊗E_{\gamma_{1}}⊗E_{\gamma_{2}}⊗E_{\gamma_{3}}⊗E_{\gamma_{4}}⊗E_{\gamma_{5}}⊗E_{\gamma_{6}}$ is a linear code of length (α+3β+6γ) over $F_{q}$, where $A_{\alpha },B_{\beta_{i}}$ and $E_{\gamma_{j}}$ are linear codes of length α,β and γ over $F_{q},P$ and Q, respectively, for i=1,2,3 and j=1,2,3,4,5,6.*

**Proof. **  The proof directly follows from the definition of a Gray map φ. □

**Theorem 13.**  *Let E be a $F_{q}PQ$-linear code of length (α+β+γ). If $A_{\alpha }^{⊥}\subseteq A_{\alpha },B_{\beta_{i}}^{⊥}\subseteq B_{\beta_{i}}$ and $(E_{\gamma_{j}}^{⊥}\subseteq E_{\gamma_{j}}$ for i=1,2,3, j=1,2,3,4,5,6. Then, there exists a quantum error-correcting code having parameters $\left[\left[\alpha +3\beta +6\gamma ,2k-\left(\alpha +3\beta +6\gamma \right),d_{H}\right]\right]$, where $d_{H}$ is the Hamming distance.*

**Proof. **  The proof is similar to that in [25]. □

## 6. Applications

In this section, we mainly focus on the applications of separable $F_{q}PQ$-cyclic codes. Using the Gray images of cyclic codes over *P*, we obtain a number of optimal linear codes in Table 1. Additionally, we describe several quantum codes over $F_{q},P,Q$, $F_{q}P$ and $F_{q}PQ$. In Table 2, Table 3, Table 4, Table 5 and Table 6, we obtain MDS quantum codes, better quantum codes than the existing codes that appeared in some reference (see [25,31,32,33,34,35] for details) and new quantum codes, respectively. The Magma computation system [36] is used to complete all of the computations in these examples and tables, and we take $\alpha_{1}=\alpha_{2}=1$ in the rings *P* and *Q*.

The invertible matrices *A* used to construct the quantum codes are as follows:
$A_{11}=\left[\begin{array}{ccc} 2 & 3 & 4\\ 2 & 1 & 2\\ 1 & 2 & 3 \end{array}\right]\in GL_{3}\left(F_{5}\right)$$A_{12}=\left[\begin{array}{ccc} 2 & 1 & 2\\ 5 & 2 & 1\\ 1 & 2 & 5 \end{array}\right]\in GL_{3}\left(F_{7}\right)$$A_{13}=\left[\begin{array}{ccc} 9 & 2 & 1\\ 10 & 9 & 2\\ 2 & 1 & 2 \end{array}\right]\in GL_{3}\left(F_{11}\right)$$A_{14}=\left[\begin{array}{ccc} 2 & 1 & 2\\ 15 & 2 & 1\\ 1 & 2 & 15 \end{array}\right]\in GL_{3}\left(F_{17}\right)$$A_{21}=\left[\begin{array}{cccccc} 2 & 3 & 4 & 0 & 0 & 0\\ 2 & 1 & 2 & 0 & 0 & 0\\ 1 & 2 & 3 & 0 & 0 & 0\\ 0 & 0 & 0 & 2 & 3 & 4\\ 0 & 0 & 0 & 2 & 1 & 2\\ 0 & 0 & 0 & 1 & 2 & 3 \end{array}\right]\in GL_{6}\left(F_{5}\right)$$A_{22}=\left[\begin{array}{cccccc} 2 & 1 & 2 & 0 & 0 & 0\\ 5 & 2 & 1 & 0 & 0 & 0\\ 1 & 2 & 5 & 0 & 0 & 0\\ 0 & 0 & 0 & 2 & 1 & 2\\ 0 & 0 & 0 & 5 & 2 & 1\\ 0 & 0 & 0 & 1 & 2 & 5 \end{array}\right]\in GL_{6}\left(F_{7}\right)$

**Example 2.**  *β=4, $\alpha_{2}=1$, q=5, and $P=F_{5}\left[v\right]/\langle v^{3}-v\rangle$.*

*Then,*

$$\kappa^{4}-1=\left(\kappa +1\right)\left(\kappa +2\right)\left(\kappa +3\right)\left(\kappa +4\right)\in F_{5}\left[\kappa \right].$$

*take $h_{1}\left(\kappa \right)=\kappa^{2}+1$, $h_{2}\left(\kappa \right)=h_{3}\left(\kappa \right)=\left(\kappa +1\right)$. Also, we let*

$$A_{11}=\left[\begin{array}{ccc} 2 & 3 & 4\\ 2 & 1 & 2\\ 1 & 2 & 3 \end{array}\right]$$

*such that $A_{1}A_{1}^{T}=4I_{3\times 3}$, where $I_{3\times 3}$ is an identity matrix of order 3. Then, $B_{\beta }$ be a cyclic code of length 4 over P and Gray image $\varphi_{P}\left(B_{\beta }\right)$ having the parameters ${\left[12,8,4\right]}_{5}$ which is an optimal code as per the database [37].*

**Example 3.**  *Let $q=5,\;\alpha_{1}=\alpha_{2}=1,\alpha =10,\;\beta =10,\;\gamma =10$ and $Q_{\alpha +\beta +\gamma }=\frac{F_{5}\left[\kappa \right]}{\left(\kappa^{10}-1\right)}\times \frac{P[\kappa ]}{\left(\kappa^{10}-1\right)}\times \frac{Q[\kappa ]}{\left(\kappa^{10}-1\right)}$, where $P=F_{5}\left[v\right]/\langle v^{3}-v\rangle$ and $Q=F_{5}\left[u,v\right]/\langle u^{2}-1,v^{3}-v\rangle$. Now, $\kappa^{10}-1={\left(\kappa +1\right)}^{5}{\left(\kappa +4\right)}^{5}\in F_{10}\left[\kappa \right]$. We take $a\left(\kappa \right)={\left(\kappa +1\right)}^{2}\left(\kappa +4\right)\in F_{5}\left[\kappa \right]$ and $A_{\alpha }=\langle a\left(\kappa \right)\rangle$ be a cyclic code of length 10 over $F_{5}\left[\kappa \right]$ with the parameters ${\left[10,7,3\right]}_{5}.$ Again, $\kappa^{10}-1={\left(\kappa +1\right)}^{5}{\left(\kappa +4\right)}^{5}\in F_{5}\left[\kappa \right].$ We take $h_{1}\left(\kappa \right)={\left(\kappa +1\right)}^{2}\left(\kappa +4\right)$, $h_{2}\left(\kappa \right)=\left(\kappa +1\right),\;h_{3}\left(\kappa \right)=1$. Let*

$$A_{11}=\left[\begin{array}{ccc} 2 & 3 & 4\\ 2 & 1 & 2\\ 1 & 2 & 3 \end{array}\right]$$

*such that $A_{1}A_{1}^{T}=4I_{3\times 3}$, where $I_{3\times 3}$ is an identity matrix of order 3. Then, $B_{\beta }$ is a cyclic code of length 10 on P and its Gray image have the parameters ${\left[30,26,3\right]}_{5}$ over $F_{5}$. Next, let us consider that $\kappa^{10}-1={\left(\kappa +1\right)}^{5}{\left(\kappa +4\right)}^{5}\in F_{5}\left[\kappa \right]$. We take $\ell_{1}\left(\kappa \right)=\ell_{4}\left(\kappa \right)={\left(\kappa +1\right)}^{2}\left(\kappa +4\right)\in F_{5}\left[\kappa \right]$, $\ell_{3}\left(\kappa \right)=\ell_{5}\left(\kappa \right)=\left(\kappa +1\right)$ and $=\ell_{2}\left(\kappa \right)=\ell_{6}\left(\kappa \right)=1$. Take*

$$A_{21}=\left[\begin{array}{cccccc} 2 & 3 & 4 & 0 & 0 & 0\\ 2 & 1 & 2 & 0 & 0 & 0\\ 1 & 2 & 3 & 0 & 0 & 0\\ 0 & 0 & 0 & 2 & 3 & 4\\ 0 & 0 & 0 & 2 & 1 & 2\\ 0 & 0 & 0 & 1 & 2 & 3 \end{array}\right]$$

such that $A_{1}A_{1}^{T}=4I_{6\times 6}$, where $I_{6\times 6}$ is an identity matrix of order 6. Then, $E_{\gamma_{j}}$ be the cyclic code of length 10 over Q and its Gray image has the parameters ${\left[60,52,3\right]}_{5}$ over $F_{5}$. Then, φ(E) is a linear code having the parameters ${\left[100,85,3\right]}_{5}$ over $F_{5}$. It is clear that, $\kappa^{10}-1\equiv \;0\;mod\left(h_{i}\left(\kappa \right)h_{i}^{*}\left(\kappa \right)\right),$ where i=1,2,3. Also, $\kappa^{10}-1\equiv \;0\;mod\left(\ell_{j}\left(\kappa \right)\ell_{j}^{*}\left(\kappa \right)\right),$ where j=1,2,3,4,5,6. With the help of Lemma 5, we have

$$\begin{array}{ccc} A_{\alpha }^{⊥} & \subseteq & A_{\alpha };\\ B_{\beta_{i}}^{⊥} & \subseteq & B_{\beta_{i}};\\ E_{\gamma_{j}}^{⊥} & \subseteq & E_{\gamma_{j}}. \end{array}$$

*By using the Theorem 13, there exists a quantum error-correcting code with the parameters ${\left[\left[100,70,3\right]\right]}_{5}$. This is a new quantum code according to the database [38].*

In Table 1, we obtain the optimal linear codes. In Table 2, we obtain MDS quantum error-correcting codes over $F_{q}$, and in Table 3 and Table 4, we obtain better quantum error-correcting codes than previously known quantum error-correcting codes. In Table 5 and Table 6, we obtain new quantum error-correcting codes over $F_{q}P$ and $F_{q}PQ$.

## 7. Conclusions

In this work, cyclic codes over $F_{q}PQ$ are introduced, where $P=\frac{F_{q}\left[v\right]}{\langle v^{3}-\alpha_{2}^{2}v\rangle }$ and $Q=\frac{F_{q}\left[u,v\right]}{\langle u^{2}-\alpha_{1}^{2},v^{3}-\alpha_{2}^{2}v\rangle }$ are nonchain finite rings and $\alpha_{i}$ are in $F_{q}/\left\{0\right\}$ for i∈{1,2}, $q=p^{m}$ with m≥1 a positive integer and *p* is an odd prime. We reviewed some characteristics of $F_{q}PQ$-cyclic codes and defined a Gray map over $F_{q}PQ$. As an application, we constructed quantum error-correcting (QEC) codes using $F_{q}PQ$-cyclic codes. This analysis can be applied to the product of finite rings in general.

## Figures and Tables

**Table 1:** Gray images of cyclic codes over *P*.

*n*	$h_{1}\left(\kappa \right)$	$h_{2}\left(\kappa \right)$	$h_{3}\left(\kappa \right)$	$\varphi_{P}\left(C\right)$	Remarks
4	$\left(\kappa^{2}+1\right)$	κ+1	κ+1	${\left[12,8,4\right]}_{5}$	optimal
5	${\left(\kappa +4\right)}^{3}$	κ+4	κ+4	${\left[15,10,4\right]}_{5}$	optimal
6	$\left(\kappa^{2}+\kappa +1\right)$	κ+1	κ+1	${\left[18,14,4\right]}_{5}$	optimal
7	$\kappa^{6}+\kappa^{5}+\kappa^{4}+\kappa^{3}+\kappa^{2}+\kappa +1$	κ+4	κ+4	${\left[21,13,4\right]}_{5}$	*…*
8	$\left(\kappa +2\right)\left(\kappa^{2}+2\right)$	κ+2	κ+1	${\left[24,19,4\right]}_{5}$	optimal
10	$\left(\kappa +1\right){\left(\kappa +4\right)}^{2}$	κ+1	1	${\left[30,26,3\right]}_{5}$	optimal
12	$\left(\kappa +4\right)\left(\kappa^{2}+2\kappa +2\right)$	κ+1	κ+1	${\left[36,31,4\right]}_{5}$	optimal

**Table 2:** MDS Quantum codes over $F_{q}$.

α	α(κ)	Parameters of $A_{\alpha }$	Quantum Codes	Remarks
3	(κ+2)	${\left[3,2,2\right]}_{3}$	${\left[\left[3,1,3\right]\right]}_{3}$	MDSCode
4	(κ+1)(κ+2)	${\left[4,2,3\right]}_{5}$	${\left[\left[4,0,3\right]\right]}_{5}$	MDSCode
5	${\left(\kappa +4\right)}^{2}$	${\left[5,3,3\right]}_{5}$	${\left[\left[5,1,3\right]\right]}_{5}$	MDSCode
6	$\left(\kappa +1\right)\left(\kappa^{2}+\kappa +1\right)$	${\left[6,3,4\right]}_{5}$	${\left[\left[6,0,4\right]\right]}_{5}$	MDSCode
7	${\left(\kappa +6\right)}^{2}$	${\left[7,5,3\right]}_{7}$	${\left[\left[7,3,3\right]\right]}_{7}$	MDSCode
7	${\left(\kappa +6\right)}^{3}$	${\left[7,4,3\right]}_{7}$	${\left[\left[7,1,4\right]\right]}_{7}$	MDSCode
8	$\left(\kappa +1\right)\left(\kappa^{2}+3\kappa +1\right)$	${\left[8,5,4\right]}_{7}$	${\left[\left[8,2,4\right]\right]}_{7}$	MDSCode

**Table 3:** Quantum codes over *P*.

β	$h_{1}\left(\kappa \right)$	$h_{2}\left(\kappa \right)$	$h_{3}\left(\kappa \right)$	Gray Image over *P*	Quantum Codes	Known Quantum Codes
25	${\left(\kappa +4\right)}^{6}$	(κ+4)	1	[75,68,3]	${\left[\left[75,61,3\right]\right]}_{5}$	NewQuantumcode
30	${\left(\kappa +4\right)}^{2}\left(\kappa^{2}+4\kappa +1\right)$	κ+1	1	[90,85,3]	${\left[\left[90,80,3\right]\right]}_{5}$	${\left[\left[90,72,2\right]\right]}_{5}$ [32]
40	${\left(\kappa +1\right)}^{2}\left(\kappa^{2}+3\right)$	κ+1	1	[120,115,3]	${\left[\left[120,110,3\right]\right]}_{5}$	${\left[\left[120,104,2\right]\right]}_{5}$ [31]
60	${\left(\kappa +1\right)}^{2}\left(\kappa^{2}+3\kappa +4\right)$	κ+1	1	[180,175,3]	${\left[\left[180,170,3\right]\right]}_{5}$	${\left[\left[168,126,3\right]\right]}_{5}$ [25]
7	${\left(\kappa +6\right)}^{3}$	(κ+6)	κ+6	[[21,16,4]]	${\left[21,11,4\right]}_{7}$	*…*
21	${\left(\kappa +3\right)}^{3}\left(\kappa +5\right)$	κ+6	1	[63,58,3]	${\left[\left[63,53,3\right]\right]}_{7}$	NewQuantumcode
28	${\left(\kappa +1\right)}^{2}\left(\kappa^{2}+1\right)$	κ+1	1	[84,79,3]	${\left[\left[84,74,3\right]\right]}_{7}$	${\left[\left[84,72,3\right]\right]}_{7}$ [33]
35	${\left(\kappa +6\right)}^{3}\left(\kappa^{4}+\kappa^{3}+\kappa^{2}+\kappa +1\right)$	κ+6	κ+1	[105,96,4]	${\left[\left[105,87,4\right]\right]}_{7}$	${\left[\left[108,28,3\right]\right]}_{7}$ [34]
77	${\left(\kappa +6\right)}^{3}(\kappa^{10}+\kappa^{9}+\kappa^{8}+$	κ+1	1	[231,216,4]	${\left[\left[231,201,4\right]\right]}_{7}$	${\left[\left[228,196,3\right]\right]}_{7}$ [35]
	$\kappa^{7}+\kappa^{6}+\kappa^{5}+\kappa^{4}$					
	$+\kappa^{3}+\kappa^{2}+\kappa +1)$					
11	${\left(\kappa +10\right)}^{3}$	(κ+10)	(κ+10)	[33,28,4]	${\left[\left[33,23,4\right]\right]}_{11}$	NewQuantumcode
11	${\left(\kappa +10\right)}^{5}$	${\left(\kappa +10\right)}^{3}$	(κ+10)	[[33,24,6]]	${\left[\left[33,15,6\right]\right]}_{11}$	NewQuantumcode
17	${\left(\kappa +16\right)}^{3}$	(κ+16)	(κ+16)	[51,46,4]	${\left[\left[51,41,4\right]\right]}_{17}$	NewQuantumcode

**Table 4:** Quantum codes over *Q*.

γ	$\ell_{1}\left(\kappa \right)=\ell_{4}\left(\kappa \right)$	$\ell_{3}\left(\kappa \right)=\ell_{5}\left(\kappa \right)$	$\ell_{2}\left(\kappa \right)=\ell_{6}\left(\kappa \right)$	Gray Image over *P*	Quantum Codes	Known Quantum Codes
5	${\left(\kappa +4\right)}^{2}$	κ+4	1	[30,24,3]	${\left[\left[30,18,3\right]\right]}_{5}$	NewQuantumcode
8	$\left(\kappa +2\right)\left(\kappa^{2}+2\right)$	κ+2	1	[48,40,3]	${\left[\left[48,32,3\right]\right]}_{5}$	*…*
10	${\left(\kappa +1\right)}^{2}\left(\kappa +4\right)$	κ+1	1	[60,52,3]	${\left[\left[60,44,3\right]\right]}_{5}$	NewQuantumcode
14	${\left(\kappa +1\right)}^{3}\left(\kappa +6\right)$	κ+1	κ+1	[84,72,4]	${\left[\left[84,60,4\right]\right]}_{7}$	${\left[\left[84,60,4\right]\right]}_{7}$ [34]
18	$\left(\kappa +3\right)\left(\kappa^{3}+2\right)\left(\kappa^{3}+3\right)$	κ+3	κ+3	[108,90,4]	${\left[\left[108,72,4\right]\right]}_{7}$	${\left[\left[108,28,3\right]\right]}_{7}$ [34]
30	${\left(\kappa +4\right)}^{2}\left(\kappa^{2}+4\kappa +1\right)$	κ+4	1	[180,170,3]	${\left[\left[180,160,3\right]\right]}_{5}$	${\left[\left[180,156,2\right]\right]}_{5}$ [32]
35	${\left(\kappa +4\right)}^{2}\left(\kappa^{6}+\kappa^{5}+\kappa^{4}+\kappa^{3}+\kappa^{2}+\kappa +1\right)$	κ+4	1	[210,192,3]	${\left[\left[210,174,3\right]\right]}_{5}$	${\left[\left[210,150,2\right]\right]}_{5}$ [32]
38	$\left(\kappa^{3}+3\kappa^{2}+4\kappa +1\right)$	$\kappa^{3}+2\kappa +6$	1	[228,216,3]	${\left[\left[228,204,3\right]\right]}_{7}$	${\left[\left[228,196,3\right]\right]}_{7}$ [34]
42	$\left(\kappa +1\right){\left(\kappa +3\right)}^{2}$	κ+1	1	[252,244,3]	${\left[\left[252,236,3\right]\right]}_{7}$	$\left[{\left[252,212,3\right]}_{7}\right]$ [34]

**Table 5:** Quantum codes over $F_{q}P$.

α	β	α(κ)	$h_{1}\left(\kappa \right)$	$h_{2}\left(\kappa \right)$	$h_{3}\left(\kappa \right)$	Gray Image over $F_{q}P$	New Quantum Codes
10	5	${\left(\kappa +1\right)}^{2}\left(\kappa +4\right)$	${\left(\kappa +4\right)}^{2}$	κ+4	1	[25,19,3]	${\left[\left[25,13,3\right]\right]}_{5}$
15	10	$\left(\kappa^{2}+\kappa +1\right){\left(\kappa +4\right)}^{2}$	${\left(\kappa +1\right)}^{2}\left(\kappa +4\right)$	κ+1	1	[45,37,3]	${\left[\left[45,29,3\right]\right]}_{5}$
20	20	${\left(\kappa +1\right)}^{2}\left(\kappa +2\right)$	${\left(\kappa +1\right)}^{2}\left(\kappa +2\right)$	κ+1	1	[80,73,3]	${\left[\left[80,66,3\right]\right]}_{5}$
30	20	${\left(\kappa +1\right)}^{2}\left(\kappa^{2}+\kappa +1\right)$	${\left(\kappa +1\right)}^{2}\left(\kappa +2\right)$	κ+1	1	[90,82,3]	${\left[\left[90,74,3\right]\right]}_{5}$
7	7	${\left(\kappa +6\right)}^{3}$	${\left(\kappa +6\right)}^{3}$	κ+6	κ+6	[28,20,4]	${\left[\left[28,12,4\right]\right]}_{7}$
14	7	${\left(\kappa +6\right)}^{3}\left(\kappa +1\right)$	${\left(\kappa +6\right)}^{3}$	κ+6	κ+6	[35,26,4]	${\left[\left[35,17,4\right]\right]}_{7}$
28	28	${\left(\kappa +1\right)}^{3}\left(\kappa^{2}+1\right)$	${\left(\kappa +1\right)}^{3}\left(\kappa^{2}+1\right)$	κ+1	κ+1	[112,100,4]	${\left[\left[112,88,4\right]\right]}_{7}$
42	56	${\left(\kappa +1\right)}^{3}\left(\kappa +2\right)\left(\kappa +3\right)$	${\left(\kappa +1\right)}^{3}\left(\kappa +2\right)\left(\kappa +3\right)$	κ+1	κ+1	[210,198,4]	${\left[\left[210,186,4\right]\right]}_{7}$

**Table 6:** Quantum codes over $F_{q}PQ$.

α	β	γ	α(κ)	$h_{1}\left(\kappa \right)$	$h_{2}\left(\kappa \right)$	$h_{3}\left(\kappa \right)$	$\ell_{1}\left(\kappa \right)=\ell_{4}\left(\kappa \right)$	$\ell_{3}\left(\kappa \right)=\ell_{5}\left(\kappa \right)$	$\ell_{2}\left(\kappa \right)=\ell_{6}\left(\kappa \right)$	Gray Image over $F_{q}\mathrm{PQ}$	New Quantum Codes
5	5	5	${\left(\kappa +4\right)}^{2}$	${\left(\kappa +4\right)}^{2}$	κ+4	1	${\left(\kappa +4\right)}^{2}$	κ+4	1	[50,39,3]	${\left[\left[50,28,3\right]\right]}_{5}$
10	10	10	${\left(\kappa +1\right)}^{2}\left(\kappa +4\right)$	${\left(\kappa +1\right)}^{2}\left(\kappa +4\right)$	κ+1	1	${\left(\kappa +1\right)}^{2}\left(\kappa +4\right)$	κ+1	1	[100,85,3]	${\left[\left[100,70,3\right]\right]}_{5}$
10	20	30	${\left(\kappa +1\right)}^{2}\left(\kappa +4\right)$	${\left(\kappa +1\right)}^{2}\left(\kappa +2\right)$	κ+1	1	${\left(\kappa +1\right)}^{2}\left(\kappa^{2}+\kappa +1\right)$	κ+1	1	[250,233,3]	${\left[\left[250,216,3\right]\right]}_{5}$
30	20	40	${\left(\kappa +1\right)}^{2}\left(\kappa^{2}+\kappa +1\right)$	${\left(\kappa +1\right)}^{2}\left(\kappa +2\right)$	κ+1	1	${\left(\kappa +1\right)}^{2}\left(\kappa^{2}+2\right)$	κ+1	1	[330,312,3]	${\left[\left[330,294,3\right]\right]}_{5}$
7	21	14	${\left(\kappa +6\right)}^{3}$	${\left(\kappa +3\right)}^{3}\left(\kappa +5\right)\left(\kappa +6\right)$	κ+3	1	${\left(\kappa +1\right)}^{3}\left(\kappa +6\right)$	κ+1	κ+1	[154,132,4]	${\left[\left[154,110,4\right]\right]}_{7}$
14	28	35	${\left(\kappa +1\right)}^{3}\left(\kappa +6\right)$	${\left(\kappa +1\right)}^{3}\left(\kappa^{2}+1\right)$	κ+1	κ+1	${\left(\kappa +6\right)}^{3}\left(\kappa^{4}+\kappa^{3}+\kappa^{2}+\kappa +6\right)$	κ+6	κ+6	[308,279,4]	${\left[\left[308,250,4\right]\right]}_{7}$
48	84	70	$\left(\kappa +3\right)\left(\kappa^{3}+\kappa +3\right)$	${\left(\kappa +1\right)}^{2}\left(\kappa^{2}+2\right)$	κ+1	1	${\left(\kappa +1\right)}^{2}\left(\kappa^{4}+\kappa^{3}+\kappa^{2}+\kappa +1\right)$	κ+1	1	[720,697,3]	${\left[\left[720,674,3\right]\right]}_{7}$
63	56	98	${\left(\kappa +3\right)}^{2}\left(\kappa^{3}+3\right)$	${\left(\kappa +1\right)}^{2}\left(\kappa^{2}+3\kappa +1\right)$	κ+1	1	${\left(\kappa +1\right)}^{8}\left(\kappa +6\right)$	κ+1	1	[819,789,3]	${\left[\left[819,759,3\right]\right]}_{7}$

## Data Availability

Data sharing is not applicable to this article as no data sets were generated or analyzed during the current study.
